# Revisiting the reaction pathways for phospholipid hydrolysis catalyzed by phospholipase A2 with QM/MM methods†

**DOI:** 10.1039/d4sc02315c

**Published:** 2024-05-22

**Authors:** Alexandre V. Pinto, Pedro Ferreira, Ana V. Cunha, Remco W. A. Havenith, Alexandre L. Magalhães, Maria J. Ramos, Pedro A. Fernandes

**Affiliations:** a LAQV/Requimte, Departamento de Química e Bioquímica, Faculdade de Ciências da Universidade do Porto Rua do Campo Alegre, s/n 4169-007 Porto Portugal pafernan@fc.up.pt; b MolSpec, Departement Chemie, Universiteit Antwerpen Groenenborgerlaan 171 2020 Antwerpen Belgium; c Stratingh Institute for Chemistry and Zernike Institute for Advanced Materials, Rijksuniversiteit Groningen Nijenborgh 4 9747 AG Groningen Netherlands; d The Netherlands and Ghent Quantum Chemistry Group, Department of Chemistry, Ghent University Krijgslaan 281 (S3) B-9000 Gent Belgium

## Abstract

Secreted phospholipase A2 (sPLA2) is a Ca^2+^-dependent, widely distributed enzyme superfamily in almost all mammalian tissues and bacteria. It is also a critical component of the venom of nearly all snakes, as well as many invertebrate species. In non-venomous contexts, sPLA2 assumes significance in cellular signaling pathways by binding cell membranes and catalyzing ester bond hydrolysis at the sn-2 position of phospholipids. Elevated levels of GIIA sPLA2 have been detected in the synovial fluid of arthritis patients, where it exhibits a pro-inflammatory function. Consequently, identifying sPLA2 inhibitors holds promise for creating highly effective pharmaceutical treatments. Beyond arthritis, the similarities among GIIA sPLA2s offer an opportunity for developing treatments against snakebite envenoming, the deadliest neglected tropical disease. Despite decades of study, the details of PLA2 membrane-binding, substrate-binding, and reaction mechanism remain elusive, demanding a comprehensive understanding of the sPLA2 catalytic mechanism. This study explores two reaction mechanism hypotheses, involving one or two water molecules, and distinct roles for the Ca^2+^ cofactor. Our research focuses on the human synovial sPLA2 enzyme bound to lipid bilayers of varying phospholipid compositions, and employing adiabatic QM/MM and QM/MM MD umbrella sampling methods to energetically and geometrically characterize the structures found along both reaction pathways. Our studies demonstrate the higher frequency of productive conformations within the single-water pathway, also revealing a lower free energy barrier for hydrolyzing POPC. Furthermore, we observe that the TS of this concerted one-step reaction closely resembles transition state geometries observed in X-ray crystallography complexes featuring high-affinity transition state analogue inhibitors.

## Abstract 2

This study unveils hGIIA sPLA2's affinity for anionic bilayers, pinpointing crucial interfacial residues. It also emphasizes the dominance of the single-water pathway in its reaction mechanism, crucial for drug design targeting transition states.

## Graphical abstract

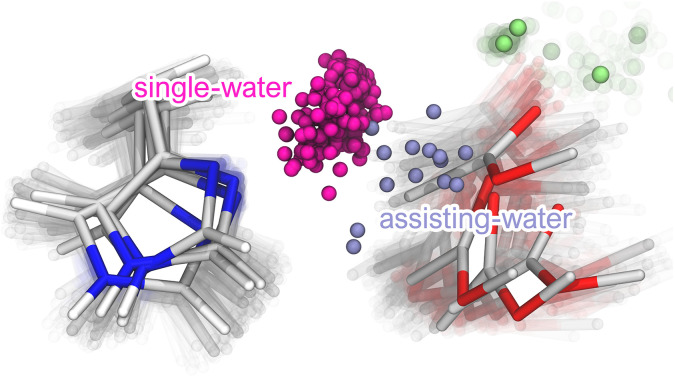

## Introduction

Secreted phospholipase A2 enzymes (sPLA2) are a diverse subgroup of the PLA2 superfamily ubiquitous across the Eukarya and Bacteria taxonomic domains, where they exert a great variety of functions.^1^ For example, human sPLA2s are involved in a myriad of essential roles that are vital for cellular homeostasis and overall physiological health.^1^ Notably, snake venom sPLA2s have been hypothesized to originate from gene duplication events,^2,3^ which lead to the accumulation of toxic variants in the venom glands of some species. Despite being related to their physiological counterparts, sPLA2 enzymes from animal venoms disrupt the normal cellular behavior of their target organisms.^4^ One well-known human isoform is often called “synovial sPLA2” due to its high concentration in the synovial fluid of patients with arthritis pathologies,^5^ such as rheumatoid arthritis and osteoarthritis. First isolated around 1989,^6,7^ it belongs to the PLA2 Group IIA (GIIA), displaying similarities to sPLA2 enzymes found in viper venoms (Type 2).^8^ Despite having crucial cellular functions, dysregulation of the hGIIA sPLA2 is associated with several pathological conditions.^9^ Certain biological roles of this sPLA2 are accomplished by the hydrolysis of ester bonds at the sn-2 position of phospholipid molecules, which produces fatty acids and lysophospholipids.^10^ The hydrolysis products act directly as lipid mediators (*e.g.*, lysophosphatidic acid) or indirectly as precursors (*e.g.*, arachidonic acid), tipping the balance of cellular signaling cascades that modulate inflammatory and immune responses.^10^

Although sPLA2 enzymes are secreted into the extracellular space, they exhibit very low activity against freely diffusing substrates in the extracellular fluid.^11,12^ Instead, the binding to membrane domains with specific characteristics prompts a conformational change or a facilitated substrate binding in a proper productive conformation (or both) that enhances the PLA2 catalytic activity by a factor of at least ∼10^3^, a phenomenon known as interfacial activation, whose molecular source is still a matter of debate.^11–15^ Furthermore, the hGIIA sPLA2 was reported to exhibit higher activity towards vesicles composed mainly of anionic phospholipids such as phosphatidylglycerol, phosphatidylserine, and phosphatidylethanolamine but showed little activity for phosphatidylcholine.^16,17^ The low affinity for zwitterionic vesicles has been attributed to the lack of tryptophan residues in the binding interface. For example, the V3,31W double mutant has an improved hydrolytic activity towards zwitterionic interfaces that is similar to that of sPLA2s from other groups known to bind phosphatidylcholine-rich vesicles, such as the human sPLA2 from groups GV and GX.^17,18^ It has also been proposed that its high positive charge at neutral pH (+14) provides electrostatic stabilization when binding anionic interfaces.^19,20^ Thus, the binding of hGIIA to phospholipid bilayers appears to result from a complex interplay between interactions of electrostatic and hydrophobic nature.^19,20^

The catalytic cycle conducted by the GIIA sPLA2 enzyme requires the presence of a Ca^2+^ cofactor that is bound to the active site by Asp_48_ and three backbone carbonyl oxygen atoms from residues His_27_, Gly_29_, and Gly_31_ (hGIIA numbering).^6^ In addition, X-ray structures with transition state analogs and mechanistic considerations show that two oxygen atoms of the substrate, namely those in the carbonyl group of the scissile ester bond and the phosphate group, also bind the Ca^2+^ cofactor. The latter may stabilize the negative charge that develops in the oxyanion near the transition state.^14^ A second Ca^2+^ binding site is also found near the catalytic cofactor (*i.e.*, <10 Å), which is thought to aid in the electrostatic stabilization of the oxyanion.^14,21^ Moreover, a catalytic His_47_-Asp_91_ dyad is positioned nearby, with hydrogen bonds from the N^δ^ atom of His_47_ to a water molecule and from the carboxylate of Asp_91_ to His_47_, Tyr_51,_ and Tyr_66_.^6^

**Scheme 1:**
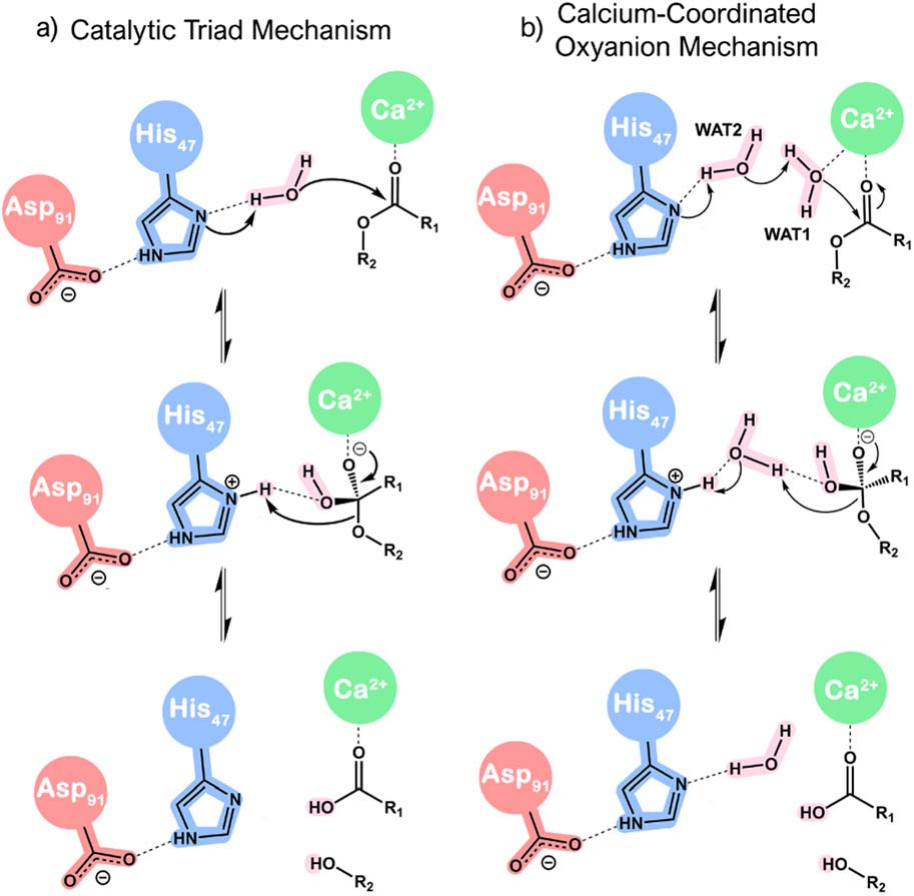
Simplified reaction mechanism proposals for sPLA2 catalysis: (a) catalytic triad mechanism and (b) calcium-coordinated oxyanion mechanism.

Two mechanistic hypotheses (Scheme 1) have been put forward: one involves a nucleophilic attack facilitated by a lone water molecule (*i.e.*, catalytic triad mechanism), while the other entails the participation of two water molecules (*i.e.*, calcium-coordinated oxyanion mechanism). For the sake of simplicity, these will be referred to in this work as the ‘single-water’ and ‘assisting-water’ pathways, respectively.^13,22–27^ The proposals present differences in terms of proton transfer paths and the role of the Ca^2+^ cofactor. The first proposal contemplates His_47_ behaving as a proton shuttle from the nucleophilic water to the product. Conversely, the other proposal assumes that protons are interchanged through WAT2 acting as a proton shuttle, donating and receiving a proton concertedly. In both cases, the Ca^2+^ cofactor polarizes the carbonyl group of the ester bond, making it more prone to nucleophilic attack and stabilizing the developing oxyanion of the tetrahedral geometry. However, an additional role appears in Scheme 1b, where Ca^2+^ is proposed to promote the formation of the nucleophile through lowering the p*K*_a_ of WAT1.

There is the possibility that the mechanistic proposals constitute competitive reaction pathways, lying close in terms of free energy of activation for the rate-limiting step and thus having comparable reaction rates.^28–30^ However, this is challenging to verify experimentally, mainly due to the inability to isolate the reaction pathways for analysis. Computational methods that rely on classical mechanics to study the conformational landscape of enzymes and quantum-mechanical (QM) or hybrid QM/MM methods to characterize the different reaction pathways accurately could shed light on the reaction mechanism of sPLA2s. However, the reported computational studies that focused on this very important problem in the past have resorted to significant approximations, such as using very small enzyme models, not including the cell membrane (known to be fundamental for the observed interfacial activation) and often describing the system with low levels of theory.^23,24,26,27^ These approximations, which were excellent for the time in which they were done, allowed for some insights into the catalytic mechanism. Nevertheless, they provided only a coarse representation of the studied phenomenon since they neglected the electrostatic environment and the conformational landscape provided by the enzyme–bilayer complex.

The hGIIA sPLA2 is a vital study case regarding the PLA2 catalytic mechanism, as it can be seen as a representative of the GI/II/V/X groups of sPLA2 enzymes.^1^ Despite a considerably low sequence identity (20–50%),^1,31^ these enzymes are structurally similar and display many conserved features such as at least six disulfide bonds (although the total number, 6 to 8, is different in each group), the catalytic His–Asp dyad, and a calcium-binding site that harbors the Ca^2+^ cofactor required for catalysis. Moreover, developing improved inhibitors for this particular sPLA2 is highly desirable for advancing new therapies for arthritis patients.^32–35^ This holds particular importance from a pharmacological perspective, considering the high prevalence of arthritis pathologies in the rapidly expanding elderly populations of developed countries, which are estimated to affect hundreds of millions around the globe.^36–38^ Insights gained from computational studies can be utilized to rationally design new inhibitors, directing the search for new drugs more efficiently and sustainably.^39^ Additionally, the similarity between human and snake sPLA2 allows to transfer the knowledge attained here to the design of new compounds for treatments of snakebite envenoming, tackling a millenary problem that still causes the death of ∼80 000 to ∼140 000 people every year.^40–42^

This study employed classical molecular dynamics (cMD) simulations to inspect the hGIIA sPLA2 enzyme bound to lipid bilayers with varying phospholipid compositions. We aimed to characterize the binding interface and interactions that are fundamental for catalysis. From the productive geometries obtained using the cMD approach, we further employed adiabatic QM/MM and QM/MM MD umbrella sampling methods to investigate different reaction pathways, describing the reactant, transition, and product states. We also compared our results with experimental findings from the literature, such as the binding poses of known inhibitors. The results clarified the details of the sPLA2-membrane binding and its reaction mechanism, which present differences and novelty relative to the current proposals. The results have a broad reach, as they might be seen as a prototype for many other secreted PLA2 from different groups and organisms.

## Methods

### Model preparation

The coordinates of the protein and calcium ions were extracted from the Protein Data Bank (code 3U8B). Hydrogen atoms were introduced using PROPKA 3.0 (ref. 43) for pH = 7.0, and final protonation states were screened with a visual inspection of the surrounding residues. A POPC (1,2-palmitoyl-oleoyl-*sn-glycero*-3-phosphocholine) substrate was created in Avogadro^44^ and docked manually in the active site using MM (GAFF2) geometry optimizations while keeping the protein frozen. The final POPC pose closely superimposes with substrate analog geometries found in the Protein Data Bank (ESI Fig. S1†). The final complex was then added to a lipid bilayer with the CHARMM-GUI server,^45^ leaving the protein at a 5 Å distance from the phospholipid headgroups. We created six systems, each with 160 phospholipids (including the substrate), and having mixed POPC : POPS compositions, that range from a pure POPC bilayer to a 1 : 1 POPC : POPS ratio. This composition range should allow to mimic different cellular conditions, since healthy cell membranes are abundant in POPC, and phosphatidylserine is exposed in the outer leaflet during apoptosis or cellular stress.^46^ The systems were then solvated, and counterions (sodium and chloride) were added to neutralize the excess charge and mimic the NaCl concentration (∼0.08–0.30 M for Na^+^ and ∼0.08–0.15 M for Cl^−^) found in the extracellular fluid within pathological contexts.^47,48^ The initial size of the simulation boxes was set as roughly 70 × 70 × 90 Å^3^, so that the minimum distance to the periodic image of the protein is larger than twice the cutoff for the short-range interactions and the real part of the long-range interactions. The protein was then parameterized with the Amber ff99SB-ILDN force field,^49^ the phospholipids with Slipids-2020,^50^ and water with TIP3P.

### Molecular dynamics simulations

All-atom molecular dynamics simulations were performed in the NPT ensemble using GROMACS 2020,^51^ at a reference temperature of 310.15 K and pressure of 1.0 bar. We used a leap-frog algorithm^52^ as the integrator, with a time-step of 2 fs. Bonds involving hydrogen were constrained with the LINCS algorithm.^53^ The electrostatic interactions were calculated with the Particle-Mesh Ewald (PME) method.^54^ We used a 10 Å cutoff for both short-range and long-range interactions, with the Lennard-Jones interactions and the real part of the electrostatic interactions being truncated beyond this value. We applied long-range dispersion corrections for the energy and pressure, and periodic boundary conditions (PBC) were applied in all directions. The temperature of the systems was controlled using a Nose–Hoover thermostat^55^ with a coupling frequency of 0.5 ps, employing two temperature coupling groups (*i.e.*, holoenzyme–bilayer complex and the rest of the system). Pressure coupling was set as semi-isotropic (*i.e.*, isotropic only in the *x* and *y* direction) using a Parrinello-Rahman barostat,^56^ with a coupling constant of 5.0 ps. The center of mass motion was removed for the holoenzyme–bilayer complex and the rest of the system separately to avoid lateral diffusion. Production runs were 200 ns each (see ESI, pages S1–S3† for a detailed description of the equilibration protocol). Using the calculated trajectories, we performed MM-PBSA calculations with the gmx_MMPBSA program^57,58^ to determine the most stable protein-bilayer complex, for which we performed two additional MD simulation replicas to increase conformational sampling. MM-PBSA calculations were performed for a temperature of 310.15 K, using an ionic strength of 0.15 M and an internal dielectric constant of 2.0. Trajectories were analyzed with the GROMACS suite of programs and the VMD 1.9.4 molecular modelling and visualization computer program.^59^

### QM/MM calculations

**Fig. 1:**
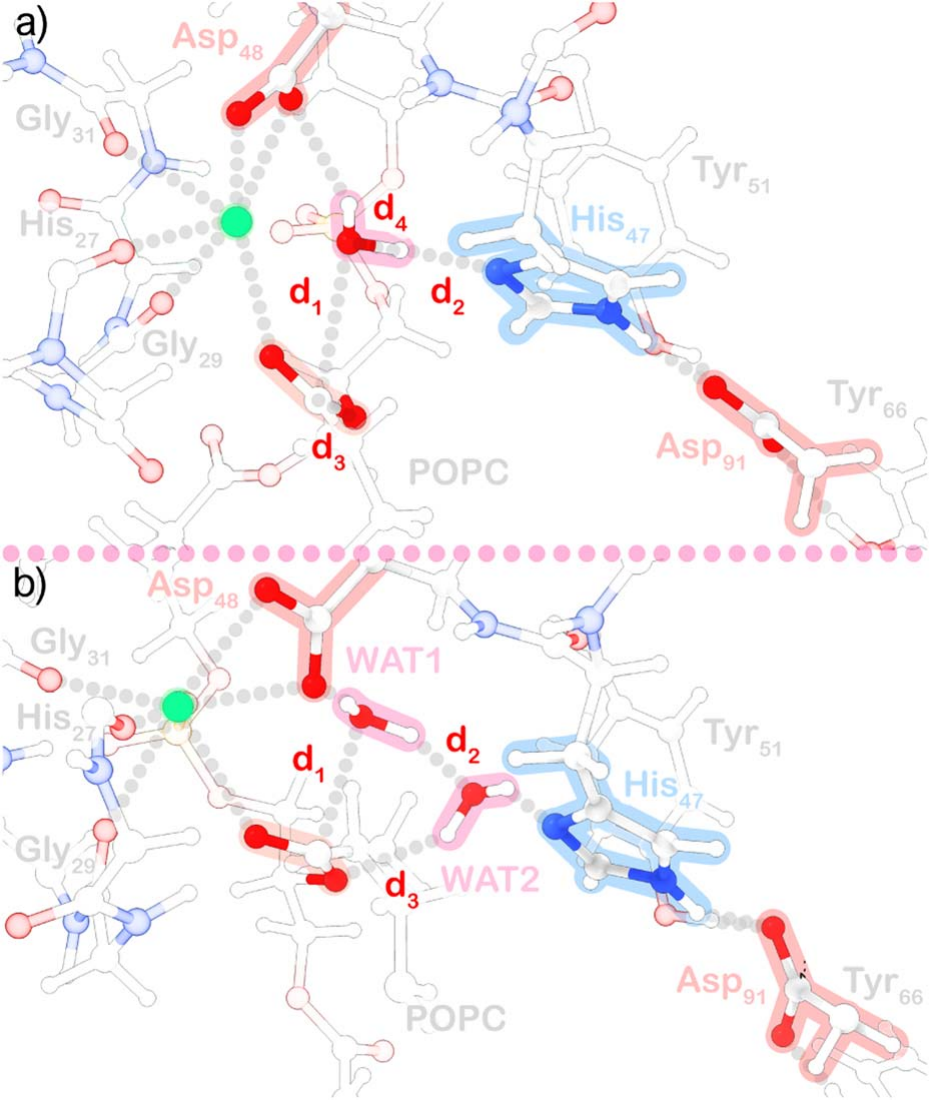
Representation of the QM region used in the (a) single-water and (b) assisting-water pathway.

All QM/MM calculations were performed in CP2K 8.2,^60^ using Density Functional Theory (DFT) with the PBE exchange–correlation functional.^61^ We employed a Gaussian plane wave (GPW) basis set^62^ of double-ζ valence polarization (DZVP-GTH-PBE) for valence electrons, together with a planewave cutoff of 300 Ry and Goedecker-Teter-Hutter (GTH-PBE) pseudopotentials^63^ for core electrons. The forces in the QM region were computed with the QUICKSTEP module,^64^ while those in the MM region were calculated with the FIST driver. The dimensions of the QM cells were 31.91 × 25.83 × 25.18 Å^3^ (164 atoms) and 36.45 × 24.42 × 23.98 Å^3^ (167 atoms), for the single-water and assisting-water systems (Fig. 1a and b), respectively, with a total charge of 0 and singlet spin multiplicity. The boundary atoms of the QM layer with dangling bonds were capped with hydrogen atoms within the IMOMM link treatment.^65^ The electrostatic coupling between the QM and MM regions was implemented through a Gaussian expansion of the electrostatic potential^66^ using the GEEP library with six Gaussian functions. The dimensions of the whole systems were 93.21 × 88.40 × 103.30 Å^3^ with PBC applied in all directions. Electrostatic interactions were computed using the smooth particle mesh Ewald method^67^ and a periodic solver for the Poisson equation. Non-bonded interactions were truncated beyond 10 Å. We employed scaling factors of 0.8333 and 0.5 for 1–4 electrostatic and van der Waals interactions. The Slipids-2020 force field, although displaying better performance to mimic NMR conformational ensembles,^68^ was replaced by the Lipids17 force field^69^ to produce a complete Amber parameterization of the MM part, considering that significant conformational changes are not expected in the picosecond timescale employed in these simulations. Parmed^70^ and cpptraj^69^ were used to process topology files and analyze trajectories, respectively.

### QM/MM nudged elastic band

For the static approach, the initial geometries were successively minimized with molecular mechanics, QM/MM with mechanical embedding, and finally, QM/MM with electrostatic embedding. The coordinates of water molecules beyond 15 Å of the protein and phospholipids 5 Å away from the substrate molecule were fixed after this stage to reduce the number of degrees of freedom. Subsequently, we performed potential energy surface scans along collective variables (CVs) comprised of distance functions to search for adequate transition state (TS) guesses that led to the formation of the products. The TS guesses were then optimized with the dimer method. The optimized TS structures were submitted to a mode selective vibrational analysis, focusing on the collective variables being analyzed, and a single negative frequency was found in both cases. Then, we used the reactant, transition state, and product geometries to run climbing image nudge elastic band (CI-NEB) calculations,^71^ which allowed us to find adiabatically connected molecular states (optimized geometries provided in the ESI†). The band scheme employed requires a projection in a subspace of collective variables to obtain a minimum energy profile. We used CV_1_ = *d*_1_ + *d*_2_ and CV_2_ = *d*_3_ + *d*_4_ for the single-water case (Fig. 1a), and CV_1_ = *d*_1_ + *d*_2_ + *d*_3_ for the two-water case (Fig. 1b), with force constants of 25 kcal mol^−1^ Å^−2^, and a spring constant of 0.05 Hartree Bohr^−2^ for the band. The geometries of end and saddle points obtained with the CI-NEB method were then refined with geometry optimizations. Atomic charges were calculated with the atomic dipole moment corrected Hirshfeld (ADCH) population method,^72^ using the Multiwfn 3.8 program.^73^ The refined molecular states were used for single-point calculations to quantify the energy contribution of MM amino acid residues to the energy barrier associated with the TS (ΔΔ*E*_a_), through amino acid residue deletion.^74^

### QM/MM MD umbrella sampling

For the dynamics approach, all Born-Oppenheimer^75^ MD simulations were carried out in the NVT ensemble, using a timestep of 1 fs and setting the canonical sampling through velocity rescaling (CSVR) thermostat^76^ with a time constant of 200 fs. First, the structures previously minimized were equilibrated in the NVT ensemble for 1 ps. The equilibrated systems were then submitted to steered-molecular dynamics (SMD) runs using the same collective variables as employed in the calculations with the static approach. Each SMD simulation had a duration of 5 ps. We applied moving harmonic restraints with a rate of approximately 0.001 Å fs^−1^ and a force constant of 200 kcal mol^−1^ Å^−2^. Geometries extracted from the SMD trajectories were used to initiate umbrella sampling^77^ simulations. Each umbrella sampling window was performed with harmonic potential restraints with force constants of 50, 100, or 150 kcal mol^−1^ Å^−2^ on the mentioned CVs. The windows were run for 12.5 ps, with the initial 2.5 ps being discarded from the analysis since they correspond to the equilibration of the systems with the introduced restraints, verified by time-block analysis of the resulting umbrella sampling histograms. The free energy profiles were obtained using the weighted histogram analysis method (WHAM),^78^ using an extraction frequency of 5 fs. The cpptraj program of the AmberTools package^69^ was used to calculate the 2D-RSMD of the umbrella sampling windows.

## Results and discussion

### Both hydrophobic and electrostatic interactions stabilize the binding of hGIIA sPLA2 to mixed POPC/POPS bilayers

Molecular dynamics simulations of the hGIIA sPLA2 bound to mixed POPC/POPS bilayers were conducted to screen the optimal electrostatic environment for the enzyme to bind to the lipid phase. Examination of protein-bilayer distances (ESI Fig. S2†) reveals that the increase in the concentration of anionic phospholipids leads to an increase in the number of stabilizing interactions of electrostatic nature. This outcome is anticipated, given the notably high positive charge of the enzyme, coupled with an increase in anionic character of the lipid bilayer interface. The atomic contacts, however, remain somewhat constant in all simulations, which suggests that hGIIA sPLA2 binding to anionic bilayers could be dominated by electrostatic interactions, namely neutral hydrogen bonds and salt bridges between positively charged amino acids and negatively charged groups from phospholipids.

**Fig. 2:**
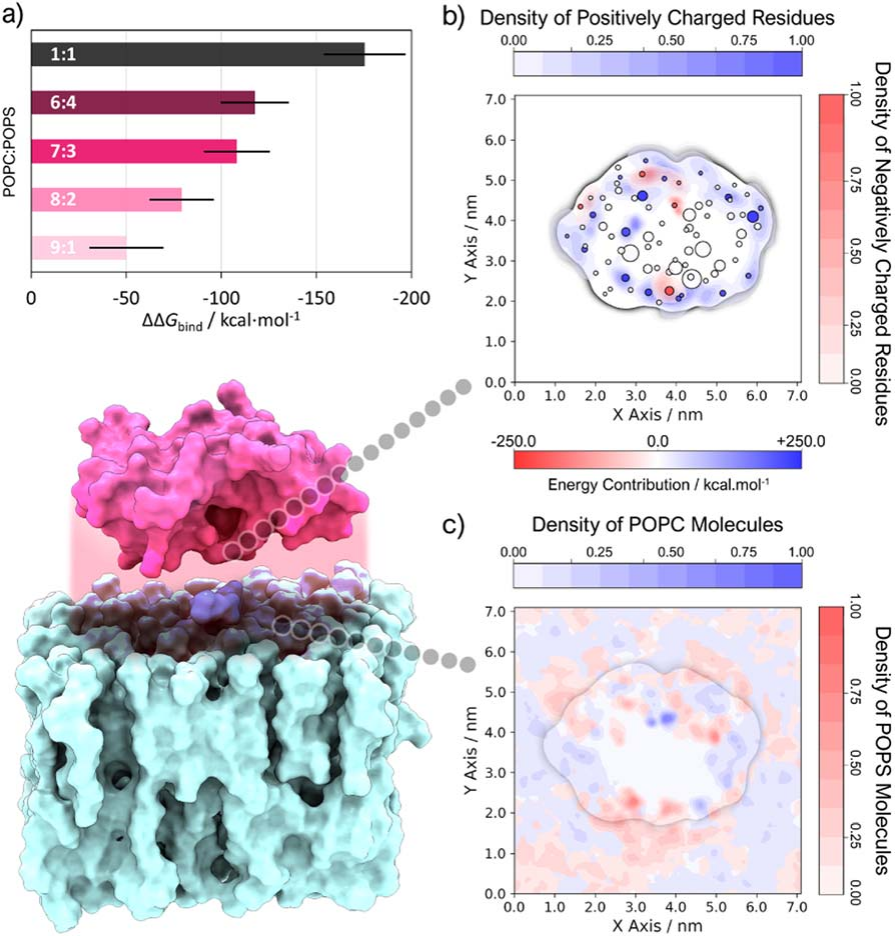
(a) ΔΔ*G*_bind_ of systems with different POPC : POPS composition, calculated relative to a pure POPC system (standard deviation is represented by a black line). (b) 2D density contour (perpendicular to the *z* axis, see ESI S4† for more details) of charged amino acid residues overlayed with a scatter plot of residues closest to phospholipid molecules, where the color and size of the markers are scaled with the energy contribution to binding (calculated with the MM-PBSA method) and the membrane penetration depth (Δ*z*) of the residue center of geometry (cog), respectively. (c) Overlay of 2D density contours (perpendicular to the *z* axis, see ESI S4† for more details) of phosphate atoms belonging to either POPC or POPS.

Indeed, MM-PBSA results (Fig. 2a) imply that GIIA PLA2 binding to lipid interfaces is significantly stabilized by a high concentration of anionic phospholipids. The binding free energy became more negative as the percentage of negative phospholipids in the mixture grew and achieved its most negative value in the 1 : 1 POPC : POPS system. This observation agrees with experimental findings that determine that a minimum percentage of *ca.* 20% negative phospholipids is needed for PLA2 binding with good affinity, and maximum affinity is reached in biomembranes with a 1 : 1 zwitterionic/anionic composition.^17^ The residue-specific energy decomposition (ESI Fig. S3†) underscores the significant stabilizing influence of positively charged residues (N-Asn, Lys, and Arg) in this context. Conversely, the limited number of negatively charged residues (C-Cys, Asp, and Glu) appears to contribute to destabilization due to repulsive interactions with POPS headgroups. Given the most favorable binding found for this last system, the altered composition of domains in cellular membranes within pathological conditions^79^ and the phospholipid concentration used in hGIIA-specific activity assays,^80^ we focus the analysis on this system from now on.

**Fig. 3:**
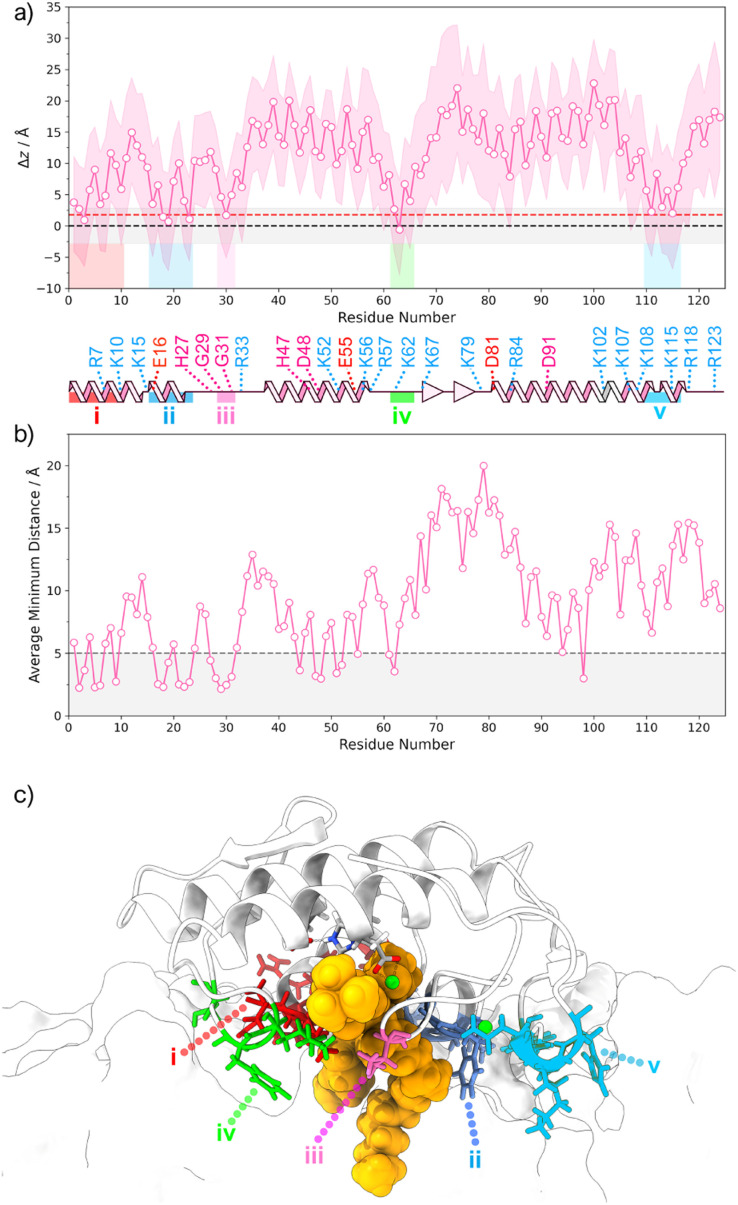
(a) Average (pink line), minimum and maximum (shaded pink band) membrane penetration depth Δ*z* of protein residues relative to the *z* axis. The membrane penetration depth is defined as the distance between the average *z* coordinates of P atoms in the upper-leaflet (*i.e.*, black dashed line) and the average *z* coordinate of the center of geometry (cog) for each residue. The standard deviation of the average *z* coordinates of P atoms in the upper-leaflet is represented as a shaded grey band, and the average *z* coordinates of headgroup atoms is represented as a red dashed line. (b) Average minimum distance between each residue and the POPC substrate molecule. The dashed line at 5 Å is a cutoff that distinguishes the closest shell of residues from the rest and was defined after a histogram analysis. (c) Representation of the residue hotspots identified to be buried deep (maximum depth is lower than the *x̄-σ* of the *z* coordinates of P atoms) in the bilayer.

The 2D density profiles of interface residues (Fig. 2b) depict a “crown” of positively charged residues (R_7_, K_10_, K_15_, R_33_, K_62_, K_67_, R_84_, K_107_, K_115_, among others) that surrounds the binding interface, which is considerably hydrophobic at the core. Interfacial residues of negative charge (E_16_, E_55_, D_81_) generally tend to be located near Lys and Arg residues, apart from Asp_48_ and Asp_91_, which are situated at the core of the protein. Except for the hydrophobic core where phosphate groups are excluded, POPC and POPS are evenly distributed throughout the simulation box (Fig. 2c), with a notable POPS density associated with the “crown” of positively charged residues. To assess penetration (ESI Fig. S4†) and identify residues submerged within the bilayer, we employed average *z*-coordinates of P atoms as a reference for calculating membrane penetration depth (Δ*z*). This measure (Fig. 3a), indicating the *z*-coordinate distance between the reference and the average center of geometry (cog) of residues, revealed five primary hotspots of buried residues located below phosphate groups: (i) N_1_, L_2_, V_3_, H_6_, R_7_, K_10_; (ii) E_16_, A_18_, L_19_, G_22_, F_23_; (iii) V_30_; (iv) K_62_, F_63_, S_65_; (v) Y_111_, S_113_, N_114_, K_115_, H_116_. These residues are commonly charged or hydrophobic, and most of them have already been identified by other authors.^17,81,82^ Hotspots (i) and (ii) are found near the POPC substrate (Fig. 3b and c) and seem to have a role in stabilizing the position of the substrate alkyl chains, while (iii) resides in the Ca^2+^ binding loop. Both hotspot (iii) and (iv) are close to the substrate's phosphate group. However, regions harboring catalytic residues are buried within the protein's core, above the line defined by the phosphate groups.

### Catalytically productive conformations of the single-water and assisting-water mechanisms coexist

**Fig. 4:**
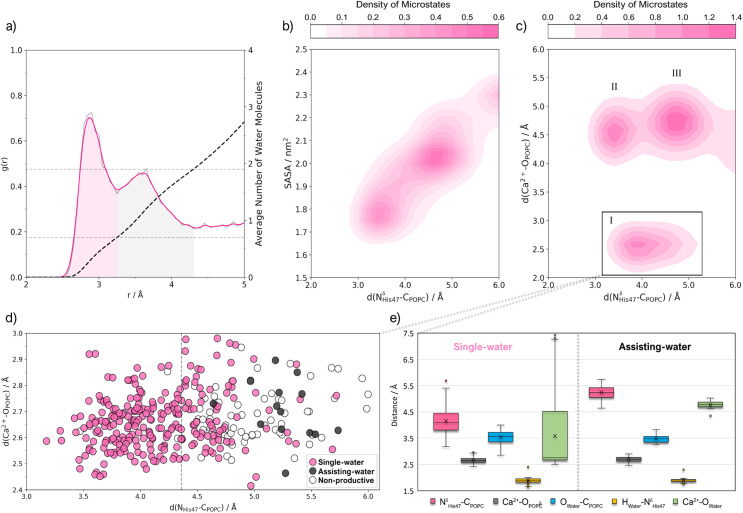
(a) Radial distribution function (RDF or *g*(*r*)), of water oxygen atoms relative to the midpoint of the N^δ^_His_-C_POPC_ vector. The grey line represents the raw data, and the pink line represents Gaussian-fitted data (*σ* = 1.5). The cumulative number of water molecules is given by the black dashed line, and the grey dashed horizontal lines indicate the average number of water molecules in each peak. (b) Contour plot of *d*(N^δ^_His_-C_POPC_) *vs.* the solvent-accessible surface area (SASA) of the N^δ^_His47_ and C_POPC_ atoms as a Gaussian-fitted density (*σ* = 1.0) of the microstates in the trajectory that display a short His_47_-water hydrogen bond. (c) Contour plot of *d*(N^δ^_His_-C_POPC_) *vs. d*(Ca^2+^-O_POPC_) as a Gaussian-fitted density (*σ* = 1.0) of microstates in the trajectory which display a short His_47_-water hydrogen bond. (d) Scatter plot of microstates from cluster I, where single-water, assisting-water and non-productive conformations have been identified with the following geometric criteria: *d*(N^δ^_His47_-H_Water_) < 2.5 Å, *d*(C_POPC_-O_Water_) < 4.0 Å and *d*(O_Water_-H_Water_) < 2.5 Å. (e) Box plot of active site distances for cluster I microstates that were identified as single-water or assisting-water conformations.

As observed in the analysis of the N^δ^_His47_-H_WAT_ distance (ESI Fig. S5†), the nitrogen atom spends nearly half of the time (∼42%) engaging in a short hydrogen bond to H atoms of water molecules. However, most of these geometries are unproductive for catalysis due to the disruption of other critical interactions (Fig. S5–S8†): the N^δ^_His47_-C_POPC_ and Ca^2+^-O_POPC_ distances are sometimes suboptimal, the catalytic water molecules are not suitably positioned near His_47_, the Asp_48_-Ca^2+^ coordination is interrupted, and the Ca^2+^ ion detaches from the binding loop. The radial distribution function (RDF) of water oxygen relative to the center of the N^δ^_His47_-C_POPC_ vector reveals two solvation spheres (Fig. 4a). The first (*ca.* 2.5 Å to 3.2 Å) displays a well-defined peak, while the second (*ca.* 3.2 Å to 4.4 Å) exhibits a broader distribution. The first solvation sphere accommodates, on average, 0.7 water molecules, whereas the second houses an average of 1.9 water molecules.

Furthermore, the instantaneous N^δ^_His47_-C_POPC_ distance, or *d*(N^δ^_His47_-C_POPC_), influences the occurrence of single-water and assisting-water conformations, serving as a sieve for water molecule diffusion. This is evident from the solvent-accessible surface area (SASA) calculations for the N^δ^_His47_ and C_POPC_ atoms (Fig. 4b), showing an increase with rising *d*(N^δ^_His47_-C_POPC_). Visual inspection of the trajectory supports this observation, with configurations resembling the single-water pathway appearing more frequently at lower *d*(N^δ^_His47_-C_POPC_) values and *vice versa* for assisting-water pathway configurations. Additionally, the RDF of oxygen atoms relative to Ca^2+^ exhibits a distinct peak up to ∼3.4 Å, corresponding to an average coordination number of 8.4 (ESI Fig. S9†). Within this solvation shell, we found at least 3.3 water molecules (ESI Fig. S10†), with the remaining oxygen atoms attributed to the sidechain of Asp_48_, the backbones of His_27_, Gly_29_, Gly_31_, the phosphate group, and, less frequently, the carbonyl oxygen of the ester bond of POPC.

Productive conformations were explored by examining the distribution of the N^δ^_His_-C_POPC_ and Ca^2+^-O_POPC_ distances within trajectory segments displaying a short His_47_-water hydrogen bond. We identified a cluster of microstates (cluster I in Fig. 4c) where catalytic distances are optimal, constituting approximately 6% of the trajectory. Two additional clusters were detected with elevated Ca^2+^-O_POPC_ distances (clusters II and III in Fig. 4c). Recognizing the significance of Ca^2+^-O_POPC_ in stabilizing the transition state, we focused on cluster I for a clustering analysis (ESI, page S8†), aiming to extract productive conformations suitable for investigating single-water and assisting-water reaction pathways. Within this cluster, we found that 75% of the microstates displayed a single catalytic water, while an assisting-water molecule was only present in 4% (Fig. 4d, ESI Fig. S11†). Moreover, most of the single-water configurations displayed the catalytic water significantly closer to the Ca^2+^ cofactor than in the assisting-water configurations. This contradicts the expectations put forward by earlier proposals.^13,22,25–27^ The single-water configurations have the catalytic water at a coordination range of Ca^2+^ (3.6 ± 1.5 Å distance) yet assisting-water configurations did not (4.8 ± 0.2 Å distance) (Fig. 4e). To select starting structures, we clustered the conformations corresponding to each pathway and analyzed the active site RMSD and catalytic distances of the representative structures of each cluster (a detailed description is given in ESI, page S8†). The chosen structures displayed a low RMSD and minimized catalytic distances, while a visual inspection ensured that the geometries were suitable for catalysis. Nevertheless, the subsequent MD sampling smoothens out much of the influence of the starting structure.

### Hydrolysis of POPC by hGIIA sPLA2 proceeds through competitive reaction pathways

**Fig. 5:**
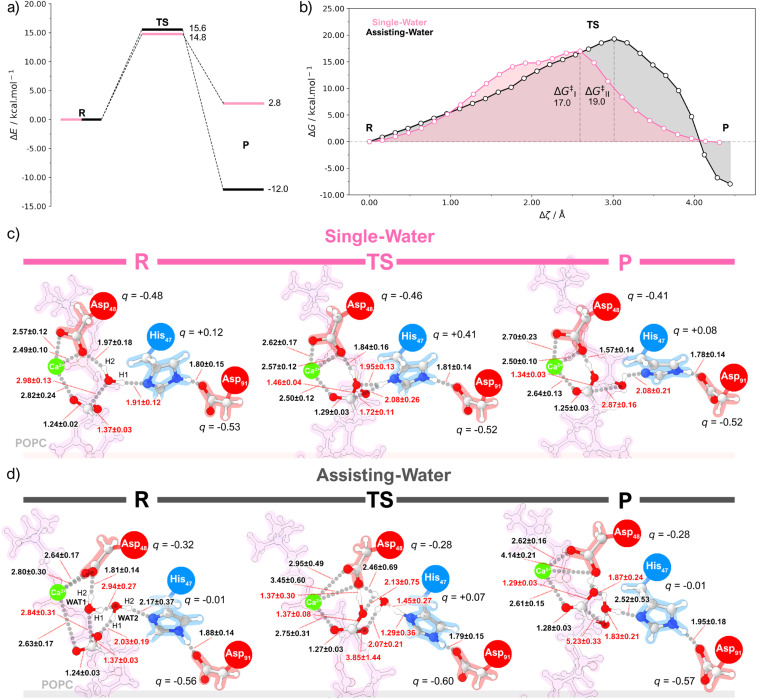
(a) Energy diagram obtained at the PBE/MM level of theory by QM/MM static calculations using the CI-NEB method for the single-water (pink) and assisting-water (black) reaction pathways. (b) Free energy curves obtained at the PBE/MM level of theory by QM/MM molecular dynamics calculations using the umbrella sampling method for the single-water (pink) and assisting-water (black) reaction pathways. Representation of chemical structures obtained with the umbrella sampling method for the (c) single-water and (d) the assisting-water mechanism.

For each pathway, the PBE/MM adiabatic CI-NEB and molecular dynamics umbrella sampling energy profiles (Fig. 5a, b, and S12†) share quite a resemblance, with a single transition state and a similar curve shape. Both methodologies suggest that the reaction pathways led to product formation in a concerted asynchronous fashion, *via* a tetrahedral oxyanion, with the transition state lying in the breakdown of this geometry (Fig. 5c and d).

For the single-water mechanism, the nucleophilic attack and subsequent formation of a tetrahedral geometry occurred concurrently with the proton transfer to His_47_ (Fig. S14, S15, S39, and S40†). The subsequent decomposition of the tetrahedral structure is initiated by the rupture of the C_POPC_–O_POPC_ bond and the repositioning of His_47_ towards the O_POPC_ atom of the scissile bond.^83^ This prompts a proton transfer from His_47_ to O_POPC_, in response to the negative charge arising on the fatty acid product. On the other hand, the assisting-water mechanism reaches a tetrahedral oxyanion *via* a nucleophilic WAT1 molecule, which is activated by the short interactions with Asp_48_ and the oxygen atom of WAT2. Subsequently, a proton transfer from WAT1 to WAT2 drives the formation of a H_3_O^+^ at the transition state for the CI-NEB method, and a H_3_O^+^-like transition state for the umbrella sampling method (ESI Fig. S23, S24, S25, S49, S50 and S51†). In the latter, the H2 proton of WAT2 and the H2 proton of WAT1 are closely shared with His_47_ and Asp_48_, respectively. At this point, the cleavage of the C_POPC_-O_POPC_ has already initiated the resolution of the tetrahedral geometry, culminating with the H1_WAT2_-O_POPC_ proton transfer.

Both Δ*E* and Δ*G*^‡^ results imply that the single-water hydrolysis has a lower activation energy, however, the calculated energy barriers are considerably close for both pathways, when considering the inherent error associated with DFT methods. From the specific activity of the GIIA sPLA2 measured using small unilamellar vesicles of 1 : 1 PLPE/POPS^80^ (*i.e.*, ∼33.9 μmol min^−1^ mg^−1^), we employed the Eyring equation, and estimated the expected free energy barrier to be near to 16.9 kcal mol^−1^. This estimate aligns closely with our umbrella sampling prediction of 17.0 kcal mol^−1^ for the activation energy of the single-water mechanism. Additionally, the 19.0 kcal mol^−1^ free energy barrier of the assisting-water pathway is reasonably close to the value determined experimentally. If one considers that the *k*_cat_ also reflects the free energy associated with the accessibility of catalytically productive conformations, then the relative populations of each reactant state calculated with MD can be incorporated into the estimate of the reaction rate.^84^ This assumes that the sampling obtained with MD is statistically representative and that the difference between free energy barriers calculated with QM/MM MD is relatively close to the ideal value. Our estimate with this approach reveals that the assisting-water pathway is about 500 times slower due to the higher free energy barrier and lower abundance of reactive conformations. The associated free energy barriers can then be obtained from the calculated rates using the Eyring equation, which results in a ΔΔ*G*^‡^ of +3.8 kcal mol^–1^ for the assisting-water pathway, relative to its single-water counterpart. The Δ*G* values calculated for the hydrolysis reaction reveal that both pathways are exergonic (*i.e.*, Δ*G*_SW_ = −0.1 kcal mol^−1^ and Δ*G*_AW_ = −7.9 kcal mol^−1^), while the product state of the assisting-water pathway is thermodynamically more favorable. This possibly results from a proton transfer from the product to Asp_48_, and stabilizing non-covalent interactions brought forward by the additional water molecule in the active site. Nevertheless, after product dissociation and active site solvation, the free energies of both pathways will necessarily become identical.

The reaction pathways resulting from the simulations present several differences concerning the current proposals in the literature: (i) the catalytic water is not coordinated to the Ca^2+^ ion, which differs from the earlier proposals, in particular for the assisting-water pathway, that assumed a Ca^2+^-bound water nucleophilic attack; (ii) the assisting-water pathway goes through a His_47_-stabilized H_3_O^+^-like transition state, rather than displaying evidence of a N^δ^_His47_-H2_WAT2_ proton transfer. The first difference might be interpreted by differentiating the roles of Ca^2+^ and Asp_48_. Mutation of Asp_48_ has been shown to completely abolish the enzyme activity, which has been interpreted in terms of impairing the binding of calcium to the active site.^85,86^ Nonetheless, our results suggest that Asp_48_ has also an active role in catalysis. In fact, the short hydrogen bonds provided by Asp_48_ (Fig. S17, S27, S44, and S55†) emphasize a role in nucleophile activation, stabilization of the transition state and lysophospholipid product in both reaction pathways. This is more noticeable in the umbrella sampling simulations of the assisting-water mechanism where proton abstractions by Asp_48_ from the newly formed carboxylic acid even occur near the transition and product states. A plausible interpretation involves the entry of water molecules by a tunnel near the choline group, which leads a path to the coordination sphere of Ca^2+^. The water molecules could subsequently be activated for catalysis by Asp_48_, possibly in tandem with the polarization introduced by the His_47_-Asp_91_ dyad.

The other disparity could be attributed to both the weak base character of His_47_ and the mobility of WAT2. Notably, the existence of short hydrogen bonds between the sidechains of Asp_91_ and Tyr_51_/Tyr_66_ may result in electron density withdrawal from the carboxylate group, which is detrimental to the stabilization of a positively charged His_47_, making it less prone to deprotonate the H_3_O^+^ ion. Additionally, the conformational lability of WAT2 can cause this molecule to abandon a productive catalytic conformation, resulting in the collapse of the tetrahedral geometry back to the reactant state (verified while probing the tetrahedral structure with QM/MM MD). Static QM/MM calculations identified a clear H_3_O^+^ transition state; a similar transition state was found with QM/MM MD, albeit with one of the H_3_O^+^ hydrogen atoms being shared closely with His_47_. The subtle geometric shifts observed between methods might be credited to the explicit conformational flexibility inherent in the umbrella sampling technique, which might lower the energy barriers for proton transfer in very short hydrogen bonds.^87^ Moreover, these findings align with the H47Q mutant displaying a vestigial catalytic activity,^22^ since the assisting-water mechanism detailed here might not require a proton abstraction by His_47_.

### hGIIA sPLA2 competitive reaction pathways rely on different electrostatic stabilization environments

Both the bond lengths and atomic dipole moment corrected Hirshfeld (ADCH) charge variations measured for structures along the CI-NEB profile hint that the single-water pathway heavily relies on His_47_ to stabilize the developing charges along the reaction path (ESI Fig. S19†). The charge of His_47_ becomes substantially more positive when the reaction coordinate is close to the tetrahedral intermediate, corresponding to the abstraction of the H1 proton. This is accompanied by a small increase in the negative charge of Asp_91_, which is stabilized by the nearby tyrosine residues (ESI Fig. S21†). The OH^−^ nucleophile transfers charge to the ester group of POPC upon bonding (ESI Fig. S20†). Since the Ca^2+^ cofactor has a filled coordination sphere and already accepts electron density from the carbonyl oxygen (ESI Fig. S22†), the excess charge is then directed towards the oxygen atom of the leaving group instead, forcing the C_POPC_-O_POPC_ bond cleavage. The accumulated charge reaches a critical point at the transition state, after which the His_47_ residue moves and donates the H1 proton to the leaving group. In the assisting-water pathway, the positive charge developed upon the H1 proton transfer gets distributed to both WAT2 and His_47_ (ESI Fig. S29†). Given that His_47_ remains unprotonated and distant from the tetrahedral intermediate, the charge lost by the OH^−^ nucleophile mirrors that received by the ester bond of POPC (ESI Fig. S30†), differing from the single-water case. The short H2_WAT1_-O1_Asp48_ hydrogen bond could also aid in concentrating the negative charge in the tetrahedral intermediate since Asp_48_ becomes remarkably less negatively charged (ESI Fig. S32†). The negative charge developed in Asp_91_ is somewhat greater here, with the tyrosine residues providing a comparatively lower degree of charge stabilization (ESI Fig. S31†).

### Identifying transition state-stabilizing residues with QM/MM methods as a strategy to guide drug design

**Fig. 6:**
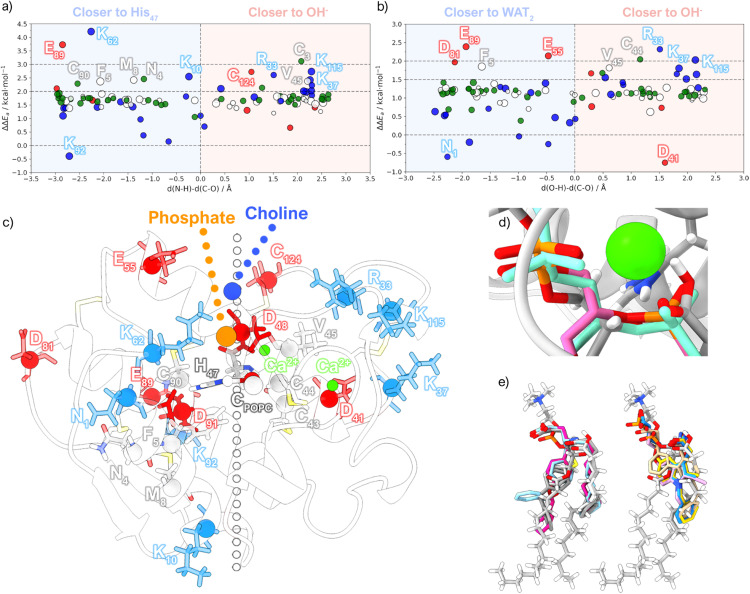
Contribution of individual residues to the energy barrier obtained from the CI-NEB calculations for the (a) single-water and (b) assisting-water reaction pathways. The energies are plotted against the *d*(N–O)-*d*(C–O) and *d*(O–H)-*d*(C–O) distance differences, respectively, which represent a plane that divides the protein into two regions: either closer to the proton-receiving base or closer to the proton donating acid. The marker color represents the amino acid classification (negatively charged – red; positively charged – blue; polar – green; apolar – white), and the marker size represents the volume. (c) Representation of the TS obtained with the single-water mechanism showing the residues identified in (a) and (b) (negatively charged – red; positively charged – blue; non-charged – grey). (d) Overlap of the single-water transition state structure obtained with the CI-NEB method (grey) and structures of a known substrate/transition-state analog present in the PDB complexes 1MKV (pink) and 1POE (aquamarine), focusing on the bond forming/breaking region. (e) Overlap of the single-water transition state structure obtained with the CI-NEB method (grey) and known inhibitors/analogs (PDB codes: 5G3N, 3U8H, 3U8D, 1KVO, 1KQU, 1J1A, 1DCY and 1DB5).

We calculated the per-residue energy contribution of the MM residues to the activation energy. The analysis also highlights differences in the electrostatic environment of the two pathways. To begin with, the energy contributions from the single-water pathway are, on average, more stabilizing than those of the assisting-water pathway (Fig. 6a and b). For instance, Glu_89_ displays an energy contribution difference of more than 1 kcal mol^−1^ in favor of the single-water pathway. Given the proximity of Glu_89_ to the catalytic His_47_, the single-water pathway greatly benefits from the stabilizing effect that the negatively charged carboxylate of Glu_89_ provides to the positive charge concentrated on His_47_. Lys_62_ also exerts significant stabilization since it is found interacting with the phosphate group of POPC. This charge neutralization effectively weakens the PO_4_^−^–Ca^2+^ interaction, increasing the net positive charge on Ca^2+^, which is advantageous to sustain the negative charge of the oxyanion. In contrast, Lys_92_ destabilizes the TS because of introduces a positive charge close to His_47_. In the assisting-water pathway, anionic residues near His_47_ or the His_47_-WAT2 moiety (*e.g.*, Glu_55_, Asp_81_, and Glu_89_) and cationic residues near the oxyanion site (*e.g.*, Arg_33_, Lys_33_, and Lys_115_) also provide substantial electrostatic TS stabilization, consistent with prior studies.^74,88^ Additionally, Asp_41_ destabilizes the TS since it is located close to the oxyanion hole, and the carboxylate is only satisfied by backbone hydrogen bonds. Meanwhile, the N-terminal Asn_1_ also destabilizes the TS because it carries a positive charge near Asp_91_, thus diverting the negative charge required to support His_47_. Finally, non-charged residues with high energy contributions (*i.e.*, Asn_4_, Phe_5_, Met_8_, Cys_43_, Cys_44_, Val_45,_ Cys_90_ and Cys_124_) are found within short non-covalent interaction distance to QM residues.

Given the multitude of degrees of freedom involved in this type of system, disparities in electrostatic stabilization for both reaction pathways could be attributed to distinct protein and bilayer conformations. These variations might create different electrostatic environments, thereby influencing the results for residues adopting alternative conformations or interacting with phospholipids carrying different charges. For instance, while Lys_62_ and Arg_33_ have been recognized to form important interactions,^17,89–92^ their identification in each case is largely attributed to the aforementioned factors. Furthermore, since the reaction pathways encompass dissimilar charge distributions in the QM region, the electrostatic contributions are also bound to vary. Thus, these results must be held as indicative of potential interaction hotspots.

Remarkably, the superimposition of calculated TS geometries with structures of transition-state-analogues from the Protein Data Bank proves to be highly accurate. As seen in Fig. 6c, the reaction region overlaps quite well with a known substrate/transition state analog. Furthermore, typical inhibitors often feature negatively charged chemical groups (*e.g.*, carboxylates) close to the phosphate group of our TS, leading to analogous Ca^2+^ coordination (Fig. 6d). The ester bond is frequently substituted by an amide bond that also coordinates Ca^2+^*via* its carbonyl group. Two distinct inhibitor types are discernible based on the core fragments housing these chemical functions. In one variant, a tertiary carbon atom resides at the core, while alkyl chains containing aromatic rings extend from this atom and the associated amide group. Surprisingly, despite the conformational freedom of the POPC substrate, the hydrophobic tails of the TS structure closely align with the ring-containing alkyl chains of these inhibitors. In contrast, the other case involves a rigid core fragment with incorporated aromatic rings, replacing the tertiary carbon, resulting in the inhibitor overlapping with only one of the hydrophobic tails of the TS.

While Lys_62_ has not been overlooked in drug design, considering that the anionic chemical groups present in the inhibitors are usually within its interaction range, our results suggest that an increase in negative charge around this region could enhance the protein–inhibitor interaction. This should be complemented by inserting a group bearing a positive charge nearby, capable of interacting with the adjacent non-conserved Glu_55_, effectively mitigating repulsive effects. Such a molecule mimics the electrostatic profile of the POPC headgroup and potentially displays prolonged residence times in the active site, coupled with enhanced specificity for hGIIA sPLA2.

## Conclusions

The molecular dynamics simulations conducted in this study have provided comprehensive insights into the binding interactions of hGIIA sPLA2 with mixed POPC/POPS bilayers of varying surface charges. According to experimental evidence in the literature,^4,79^ the abundant cationic amino acid residues dictate a preferential binding of this PLA2 to more anionic bilayer interfaces, such as bacterial cell membranes or negatively charged domains in plasma membranes (*e.g.*, phosphatidylserine exposure during apoptosis),^46^ rather than more zwitterionic interfaces. This aspect was corroborated by our molecular dynamics MM-PBSA results. The simulations further highlighted an essential role for the hydrophobic interactions, evident through residue penetration into the lipid bilayer during the molecular dynamics simulations, also in agreement with the experimental results obtained with the V3,31W double mutant.^18^ Given the limitations in the MM-PBSA method and its sensitivity to user-defined parameters, it's important to focus on understanding relative binding free energies and exercise caution when interpreting absolute binding free energies. Nevertheless, both methodologies have successfully identified critical interfacial residues (displayed in Fig. 3a), which are validated by other reports.^17,81,82^

Regarding the chemical mechanism of reaction, this study enabled the characterization of catalytically productive conformations of hGIIA sPLA2 and provided evidence supporting the kinetic feasibility of both reaction pathways, with the single-water pathway being kinetically dominant. Although the pathways resulted in products of different thermodynamic stability, both mechanisms should yield states of equal free energy by the end of the reaction cycle. The presence of two radial distribution function (RDF) peaks signifies the existence of distinct shell sizes around *d*(N^δ^_His47_-C_POPC_), each encapsulating approximately one or two water molecules. The SASA values plotted against *d*(N^δ^_His47_-C_POPC_) are consistent with this, manifesting two conformational populations characterized by distinct solvent accessibility around this distance. This solvent accessibility dependence on *d*(N^δ^_His47_-C_POPC_) has also been witnessed by other authors.^90^ The quantification of productive conformations for both reaction pathways indicates that the occurrence of the single-water reactant state is substantially more frequent, and thus carries a lower energetic penalization relative to the assisting-water counterpart.

Static and dynamic QM/MM methods revealed that the single-water pathway likely proceeds at a faster rate than the assisting-water pathway due to the lower activation free energy (14.8 *vs.* 15.6 kcal mol^−1^ using static methods and 17.0 *vs.* 19.0 kcal mol^−1^ with dynamic methods). Even though the difference is small in the face of the typical accuracy of density functional theory, the result is meaningful because the differences arise from comparing the same chemical reaction in very similar environments, a scenario that greatly benefits from error cancelation. Furthermore, the integration of both MD and QM/MM MD results in the calculation of reaction rates allowed us to estimate that the single-water pathway might be over 500 times faster. This is enforced by the energy barrier calculated for the transition state of the single-water pathway benefiting appreciably from the protein environment, as the average ΔΔ*E*_a_ results imply. Thus, the free energy profiles depicted in this study offer a unified representation of the literature proposals, portraying these as competitive parallel pathways, yet with considerably distinct reaction rates.

The electrostatic environment provided by enzymes plays a pivotal role in stabilizing transition states and reducing the free energy barrier in chemical reactions. This holds particular significance in the realm of drug design, where a successful strategy involves developing inhibitors that mimic the transition state, as enzymes lower the reaction barrier by binding more strongly to the transition state than the natural substrate. The energy contribution of each residue to the barrier provides clues on how this search can be directed.

## Data availability

Data is already provided as ESI.†

## Author contributions

A. V. P.: conceptualization, methodology, software, validation, formal analysis, investigation, writing – original draft, writing – review & editing, visualization. P. F.: writing – review & editing. A. V. C.: resources, writing – review & editing, supervision, funding acquisition. R. W. A. H.: resources, writing – review & editing, supervision, funding acquisition. A. L. M.: writing – review & editing, supervision. M. J. R.: conceptualization, writing – review & editing, supervision, funding acquisition. P. A. F.: conceptualization, writing – review & editing, supervision, funding acquisition.

## Conflicts of interest

There are no conflicts to declare.

## Supplementary Material

SC-015-D4SC02315C-s001

SC-015-D4SC02315C-s002

SC-015-D4SC02315C-s003
