# Supplementary material for: Revisiting the reaction pathways for phospholipid hydrolysis catalyzed by phospholipase A2 with QM/MM methods
Source: Chem Sci. 2024 May 22;15(25):9793–805. doi: 10.1039/d4sc02315c (PMC11206408; doi:10.1039/d4sc02315c)
Supplement: SC-015-D4SC02315C-s002 [file SC-015-D4SC02315C-s002.pdf]

Electronic Supplementary Material (ESI) for Chemical Science.
This journal is © The Royal Society of Chemistry 2024

SUPPORTING INFORMATION

"Revisiting the Reaction Pathways for Phospholipid Hydrolysis Catalyzed by Phospholipase A2 with QM/MM Methods"

1. Description of the Literature Proposals

In the single-water case, the hydrogen bond between a nearby water molecule and the His_{47} residue promotes a proton abstraction, assisted by the polarization introduced by the negatively charged Asp_{91}. Analogous to the deacylation step of the canonical serine hydrolase mechanism,[26] the resulting hydroxide nucleophile attacks the carbonyl carbon of the substrate, which gains a considerable electrophile character due to the interaction with the calcium ion. This leads to the formation of a tetrahedral intermediate. Subsequently, a proton transfer to the leaving group and the cleavage of the ester bond determine the collapse of the tetrahedral geometry.

The assisting-water alternative is relatively similar but assumes that an assisting water molecule (i.e., WAT2) receives instead a proton from the nucleophilic water molecule (i.e., WAT1), while simultaneously transferring a proton to His_{47}. The attack on the carbonyl is also ensured by an OH^- -nucleophile, which produces a tetrahedral structure. The collapse of this geometry is caused by a proton transfer from the protonated His_{47} back to the assisting water molecule, coupled with a proton transfer to the leaving group. Note that the assisting-water proposal includes coordination of the oxygen atom from the nucleophilic water to the Ca^{2+} cofactor since it could stabilize the formation of the OH^- nucleophile.[13, 20, 23, 25]

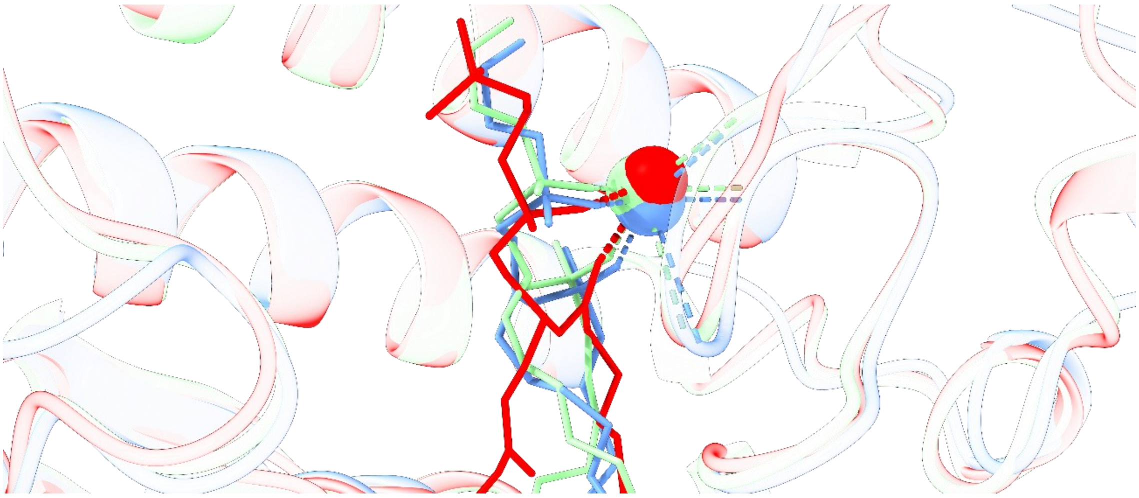

Figure S1 – Overlap between the modeled POPC molecule and crystallographic structures of complexes with L-1-O-octyl-2-heptylphosphonyl-sn-glycero-3-phosphoethanolamine (PDB codes 1MKV and 1POE).

2. Molecular Dynamics Protocol

Stage I – Membrane Equilibration

The first stage of the protocol aims to equilibrate the phospholipid bilayers, which requires a considerably long simulation time. Therefore, to avoid losing the crystallographic geometry of the enzyme, the coordinates of the PLA2-POPC complex were kept frozen (or fixed with heavy positional restraints of 10,000 $kJ.mol^{-1}$ in NPT runs) during this stage.

The systems were first submitted to a small soft-core potential minimization (maximum of 100 steps), which allowed removing bad contacts from the initial structure. After, the system was minimized using a steepest-descent algorithm with a force cutoff of 1,000 $kJ.mol^{-1}$. The system was then equilibrated in the NPT ensemble gradually by following the steps below:

1) NVT equilibration of solvent and ions, during 50 ps with a V-rescale thermostat and heavy position restraints (10000 $kJ.mol^{-1}$) on the lipids;
2) NVT equilibration of solvent, ions, and lipids, during 150 ps with a V-rescale thermostat;
3) NPT equilibration using the Berendsen thermostat and barostat during 500 ps;
4) NPT equilibration using the Nose-Hoover thermostat and the Parrinello-Rahman barostat during 500 ps;

Then, we ran a 200 ns NVT equilibration to ensure that the lipid bilayer achieved converged parameters (area-per-lipid, bilayer thickness, bilayer area, and acyl-chain order parameters), in accordance with the values found in the literature. Note that, although the holoenzyme-substrate complex was kept frozen and left at a 5 Å distance, this distance was short enough for the lipid bilayer to spontaneously accommodate the complex during this stage, avoiding the need to pull the enzyme towards the bilayer.

Stage II – Enzyme Equilibration

The second stage starts with successive minimizations (hydrogen atoms, sidechains, mainchains, and all atoms) of the atoms that were previously frozen/restrained, using a steepest-descent algorithm with a force cut-off of 1000 $kJ.mol^{-1}$. Then, we equilibrated the previously frozen/restrained atoms in the NPT ensemble:

5) NPT equilibration with the Berendsen thermostat and barostat during 500 ps and position restraints of 500 $kJ.mol^{-1}$ for the holoenzyme-substrate complex.
6) NPT equilibration with the Nose-Hoover thermostat and Parrinello-Rahman barostat during 500 ps and position restraints of 250 $kJ.mol^{-1}$ for the holoenzyme-substrate complex.

7) NPT equilibration with the Nose-hoover thermostat and Parrinello-Rahman barostat during 49 ns and harmonic potential restraints with a force constant of 1000 $kJ.mol^{-1}.nm^{-2}$ on active-site collective variables.
8) NPT equilibration with the Nose-hoover thermostat and Parrinello-Rahman barostat during 50 ns and harmonic potential restraints with a force constant of 1000 $kJ.mol^{-1}.nm^{-2}$ on catalytic collective variables.

Given that the initial holoenzyme-substrate complex geometry is considerably biased due to the modeling approach taken, we tried to minimize this by performing a pre-production run (7 and 8) where we used harmonic potentials to restrain active-site collective variables that are proposed to be essential for catalysis while keeping everything else free. The restrained distances consisted of the Asp_{91}-His_{47} hydrogen bond, $d(N_{His47}$-$C_{POPC})$, $d(Ca^{2+}$-$O_{POPC})$, and the backbone interactions of His_{27},Gly_{29} and Gly_{31} to Ca^{2+}ion. The production run was 200 ns long, without any constraints or restraints, as described in the main text.

3. Molecular Dynamics Analysis

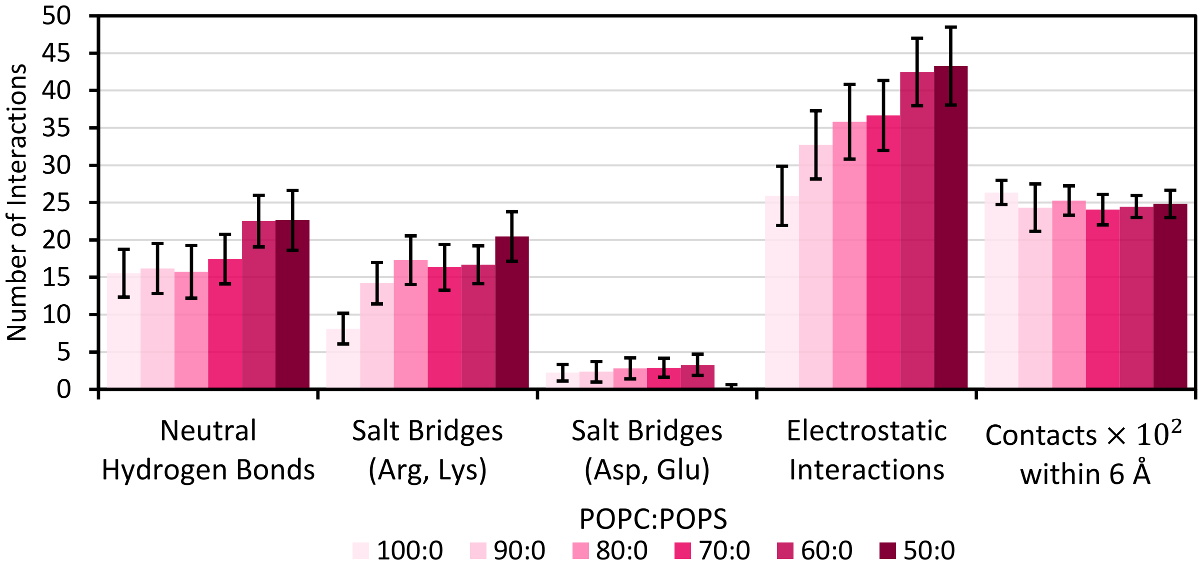

Figure S2 – Number of intermolecular interactions between the protein and the membrane bilayer. A 3.5 Å donor-acceptor distance and a 150 º hydrogen-donor-acceptor angle cutoff were used to identify neutral hydrogen bonds. Salt bridges are considered to be present when the charged groups of each pair are within 3.5 Å. This involves the phosphates from both phospholipids and the carboxylic acid groups of POPS acting as acceptors, while the choline groups from POPC and amine groups from POPS function as donors. The number of electrostatic interactions is defined as the sum of conventional hydrogen bonds and salt bridges.

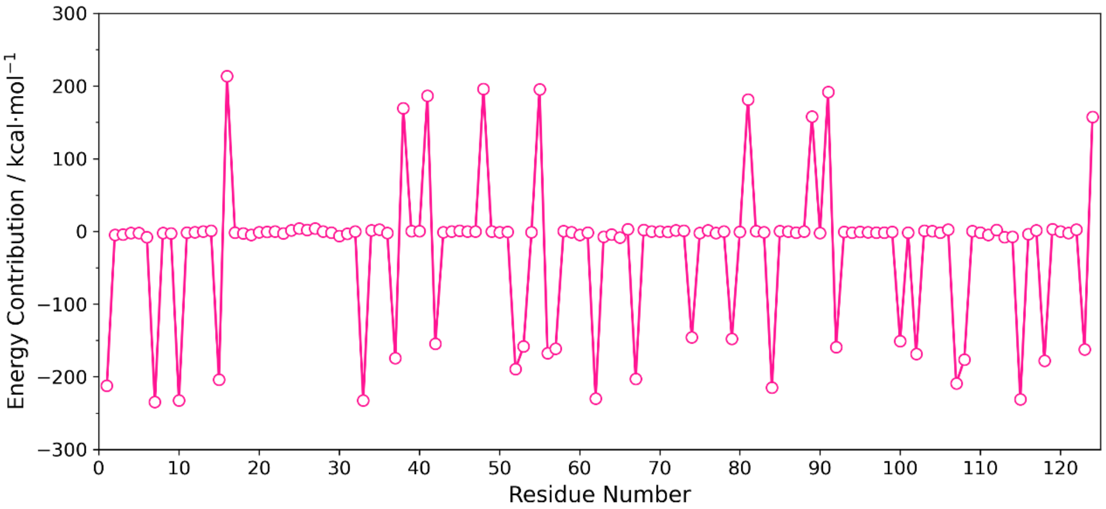

Figure S3 – PLA2-bilayer per-residue binding energy calculated with the MM-PBSA method.

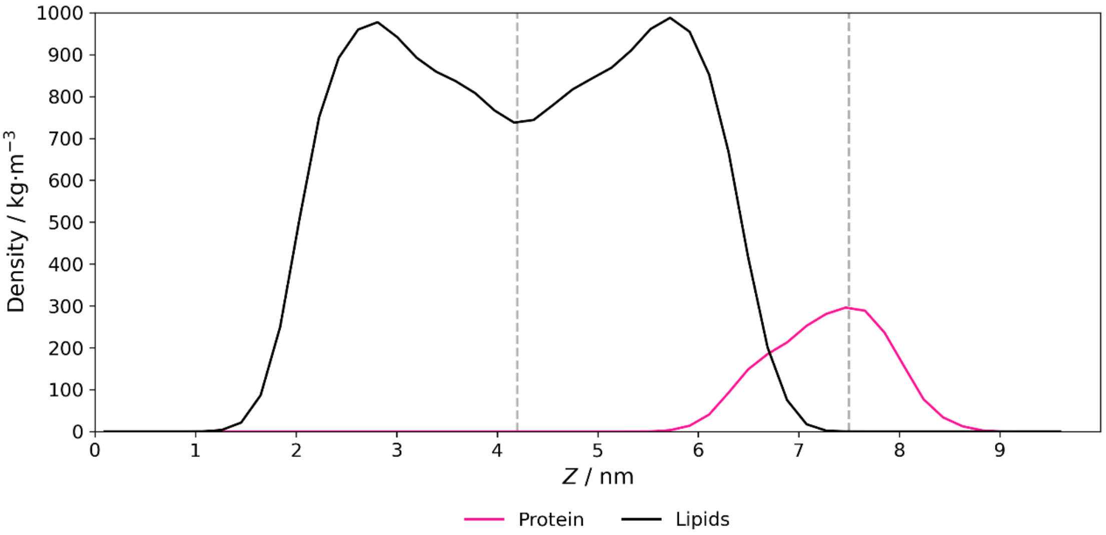

Figure S4 – Density profiles of the protein and lipids, averaged along the Z axis. Vertical dashed lines indicate the interval used for plotting 2D density profiles of the interface.

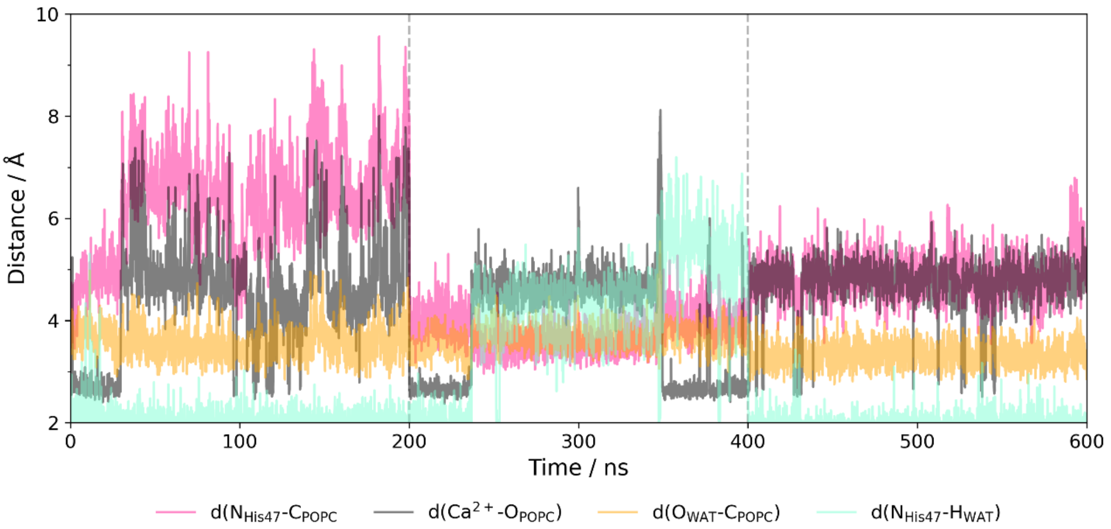

Figure S5 – Analysis of catalytic distances in the molecular dynamics simulations: between the N^{δ} atom of His_{47} and the electrophile C atom of the substrate (pink), between the Ca^{2+} cofactor and the carbonyl oxygen atom of the substrate (dark grey), between the Ca^{2+} cofactor and water O atoms (orange), and between the N^{δ} atom of His_{47} and water hydrogen atoms (aquamarine).

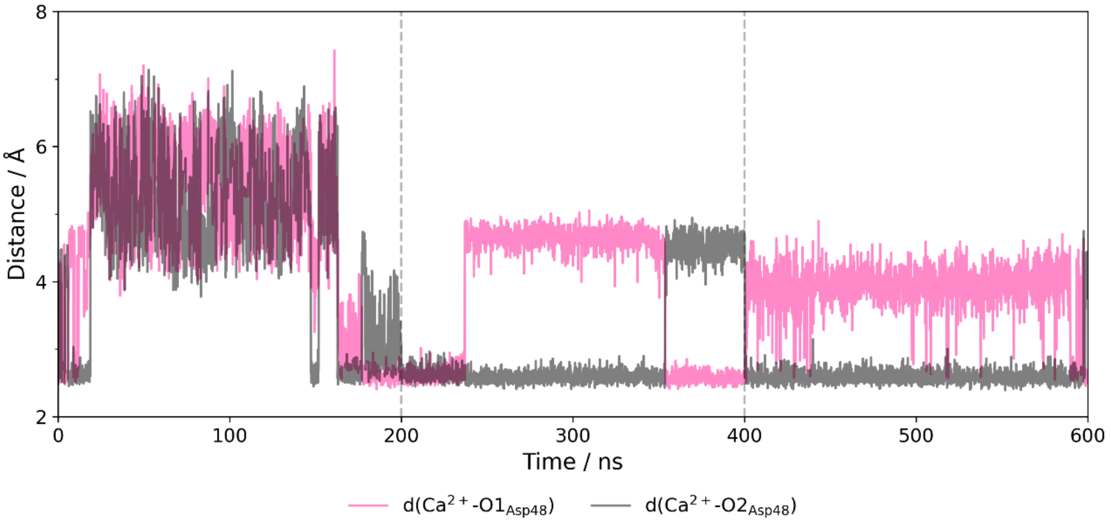

Figure S6 – Analysis of active site distances in the molecular dynamics simulations, specifically between the Ca^{2+} cofactor and the oxygen atoms of Asp_{48}.

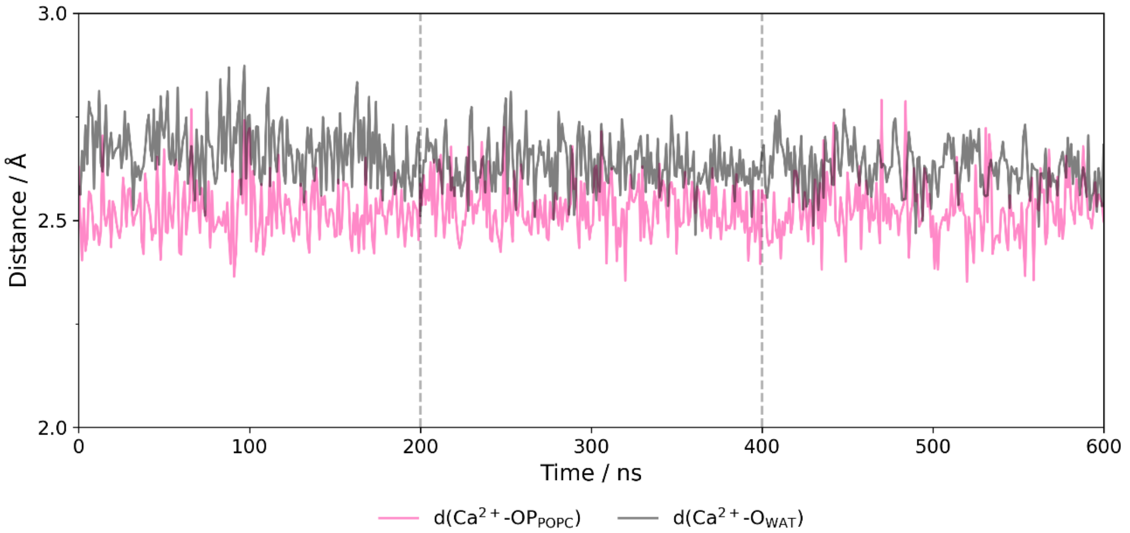

Figure S7 – Analysis of active site distances in the molecular dynamics simulations between the Ca^{2+} cofactor and the oxygen atoms of the POPC phosphate group and the Ca^{2+} cofactor and oxygen atoms of water.

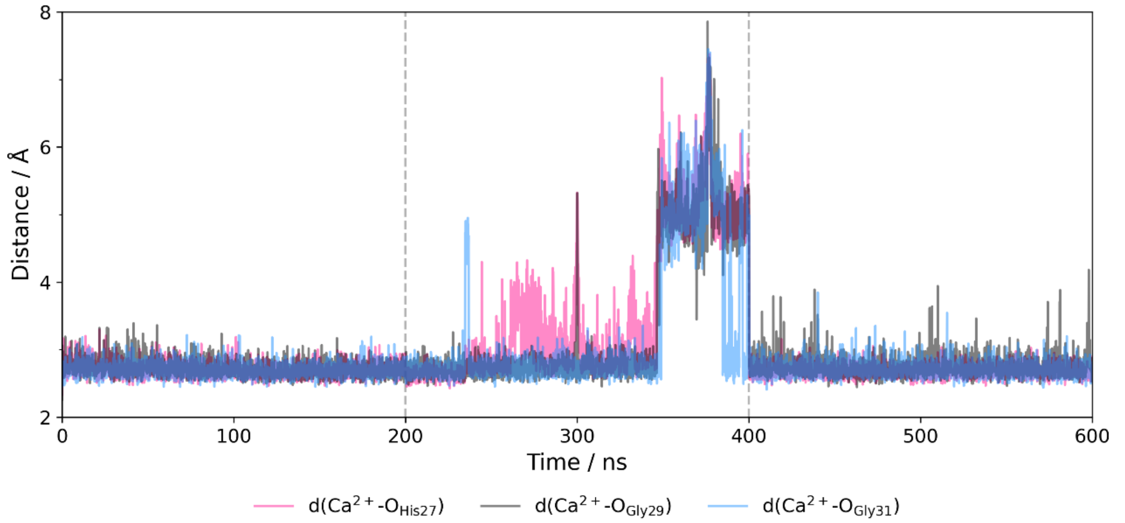

Figure S8 – Analysis of active site distances in the molecular dynamics simulations between the Ca^{2+} cofactor and the backbone oxygen atoms of the His_{27}, Gly_{29} and Gly_{31} residues.

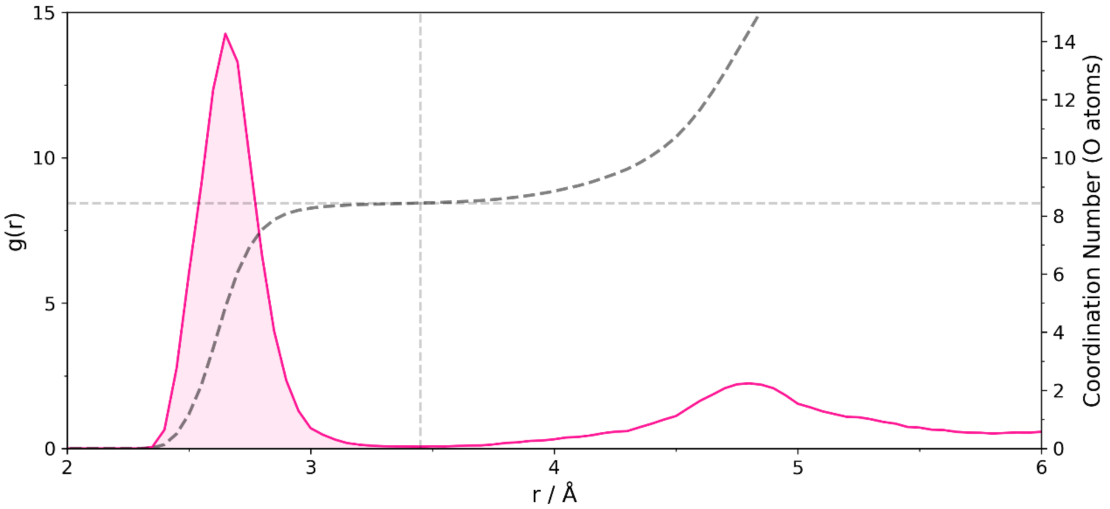

Figure S9 – Radial distribution function (RDF or g(r)) analysis of Ca^{2+} coordination by oxygen atoms. The pink line represents the RDF, and the dark grey dashed line is the cumulative number of oxygen atoms.

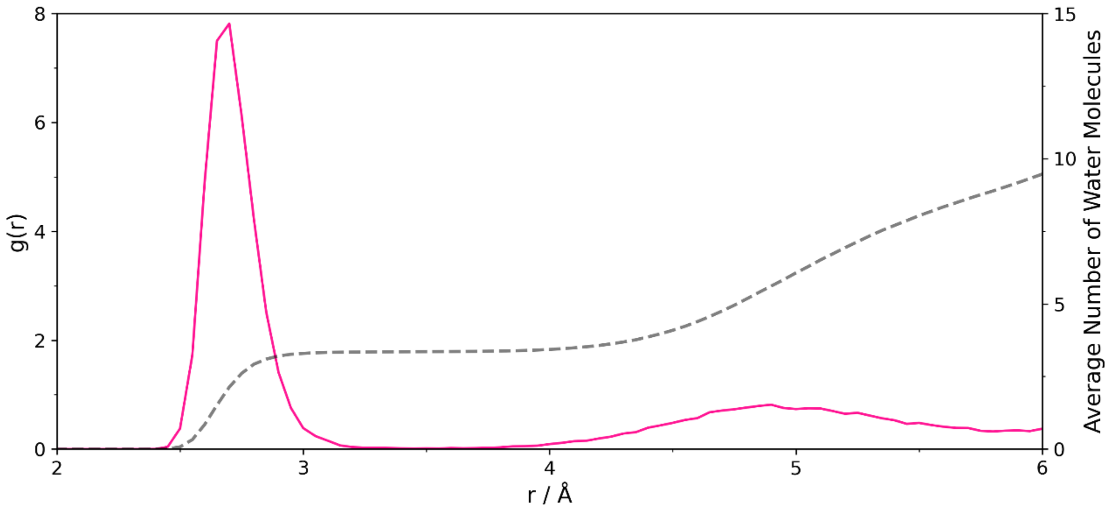

Figure S10 – Radial distribution function (RDF or g(r)) analysis of calcium coordination by water oxygen atoms. The pink line represents the RDF, and the grey dashed line is the cumulative number of water oxygen atoms.

4. Clustering Analysis

We used distances between essential catalytic interactions to screen for a pool of catalytically-productive conformations. We collected a pool of conformations where the following essential catalytic interactions are all below a pre-defined distance cutoff:

1) $d(N_{His47}\text{-}C_{POPC}) < 6.0$ Å
2) $d(Ca^{2+}\text{-}O_{POPC}) < 3$ Å
3) $d(H_{WAT\text{-}NHis47}) < 2$ Å

Then, we observed that conformations whose value of $d(N_{His47}\text{-}C_{POPC})$ is below the pool average predominantly contain a single water in the active site, while conformations whose value of $d(N_{His47}\text{-}C_{POPC})$ is above the average predominantly contain two or more water molecules. These geometries were then submitted to clustering analysis using the Gromos algorithm, with an RMSD cut-off of 0.9 Å for the region within 5 Å of the active site. We obtained 37 clusters and 46 clusters for each case, respectively.

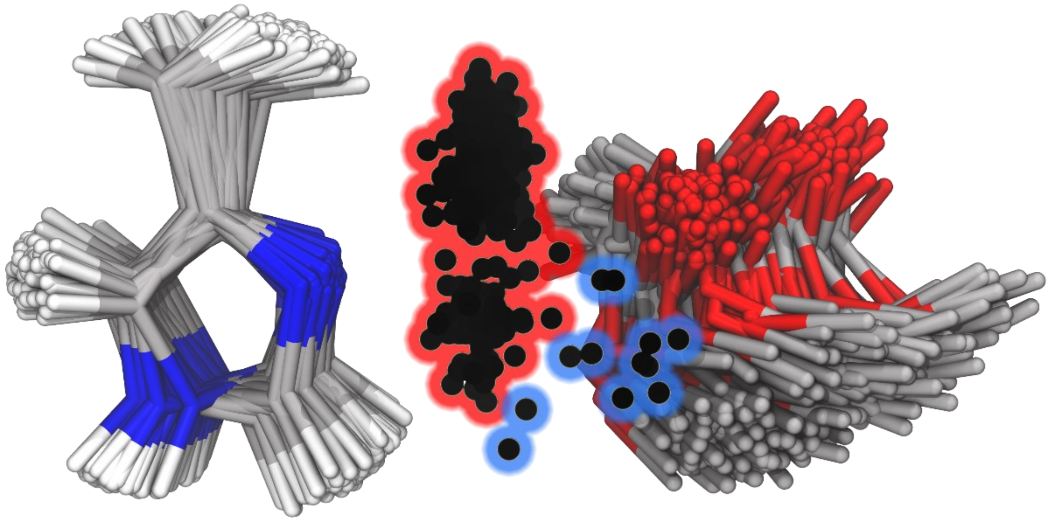

Figure S11 – Representation of the residues and water molecules involved in critical catalytic interactions in the Cluster I of microstates, where the black dots indicate the position of water O atoms. The red zone highlights water molecules hydrogen bonded to His_{47} (both reaction mechanism proposals), while the blue zone highlights adjacent waters that could participate in the assisting-water mechanism.

5. Static Calculations with the CI-NEB method

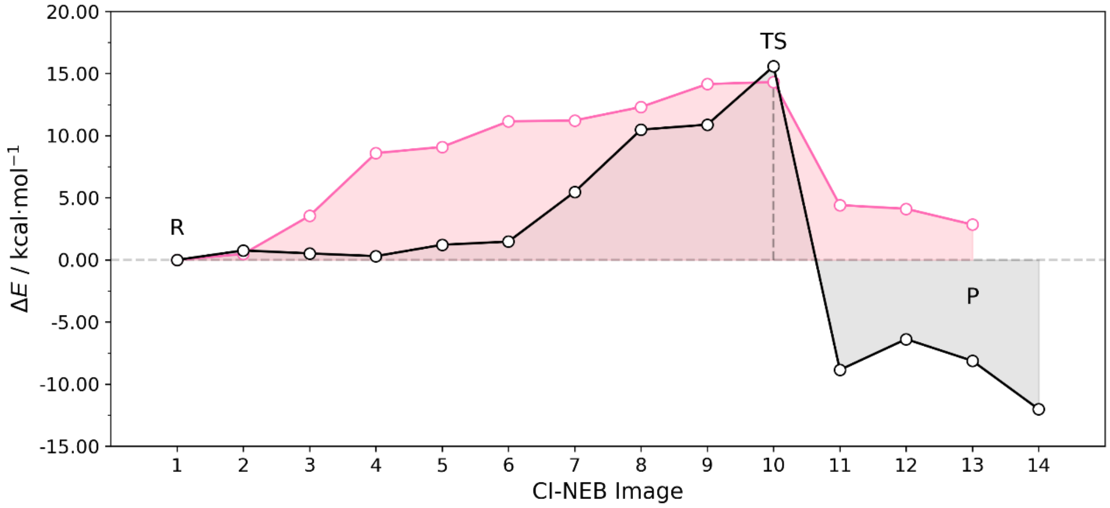

Figure S12 – Energy profiles obtained with the CI-NEB method. The pink line represents the single-water mechanism and the black line the assisting-water mechanism.

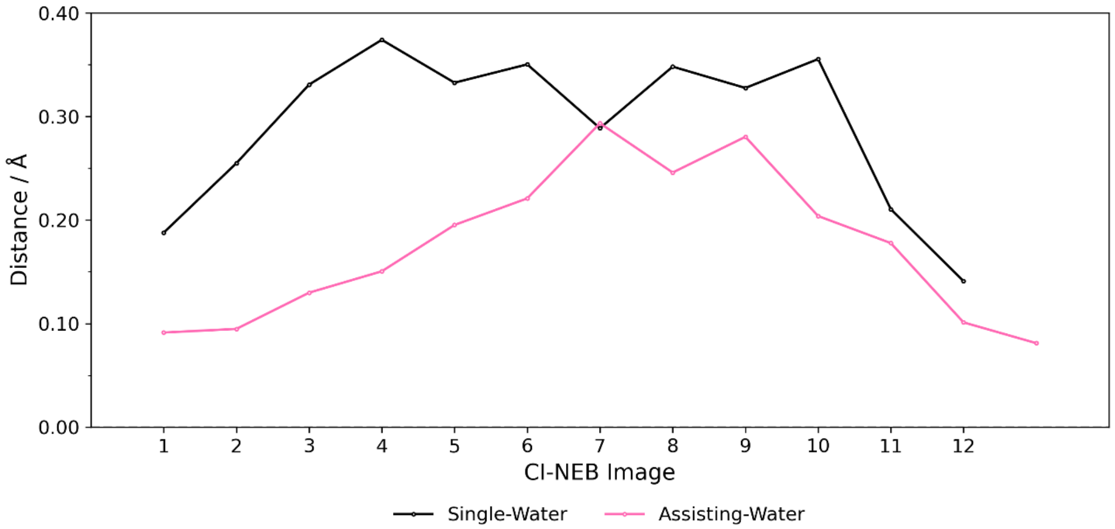

Figure S13 – Distances between successive CI-NEB images.

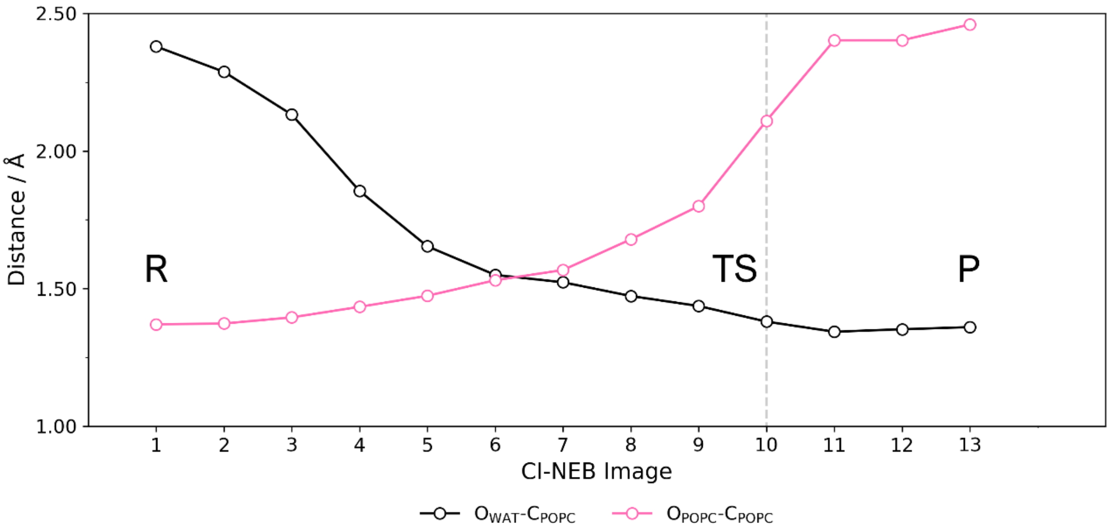

Figure S14 – Analysis of C-O bond forming (black) and C-O bond breaking (pink) distances from the CI-NEB calculations of the single-water mechanism.

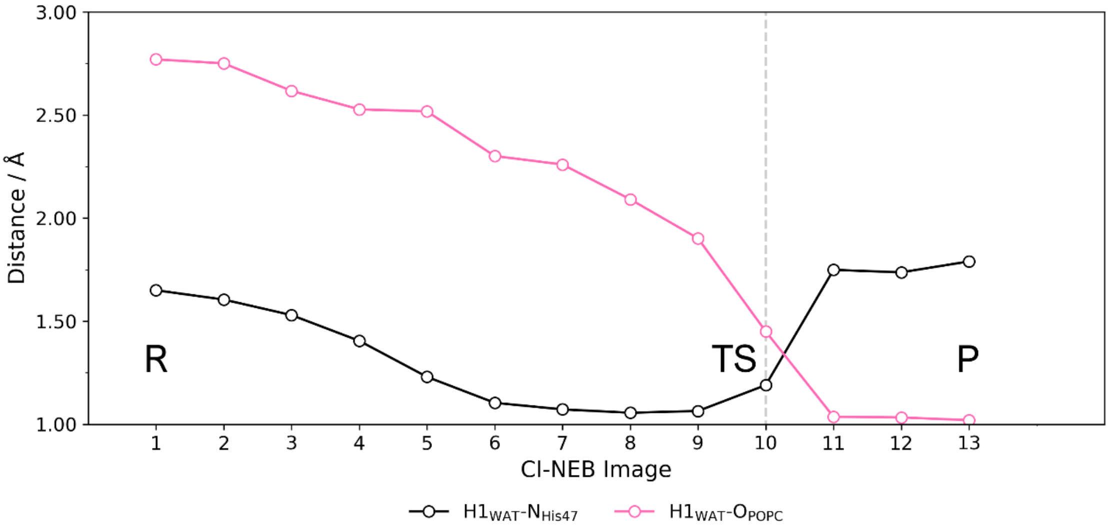

Figure S15 – Analysis of hydrogen bond forming and bond breaking distances from the CI-NEB calculations of the single-water mechanism.

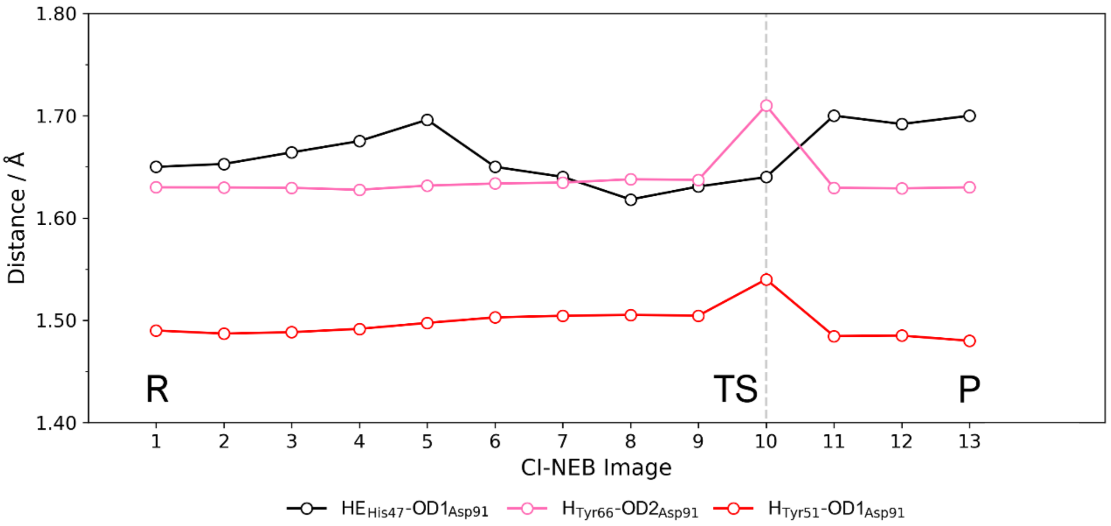

Figure S16 – Analysis of Asp_{91} hydrogen bond distances from the CI-NEB calculations of the single-water mechanism.

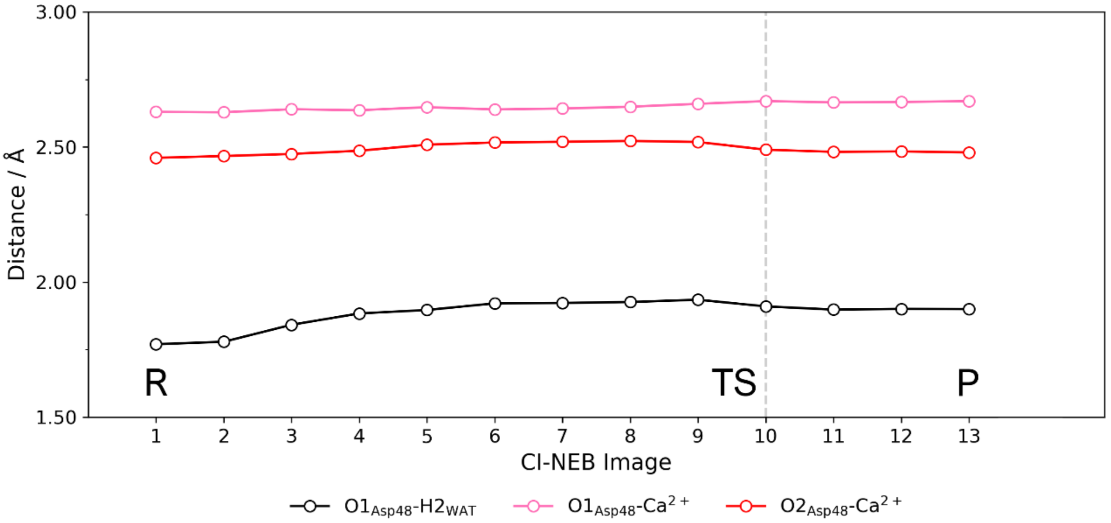

Figure S17 – Analysis of Asp_{48} distances to the Ca^{2+} cofactor and catalytic water from the CI-NEB calculations of the single-water mechanism.

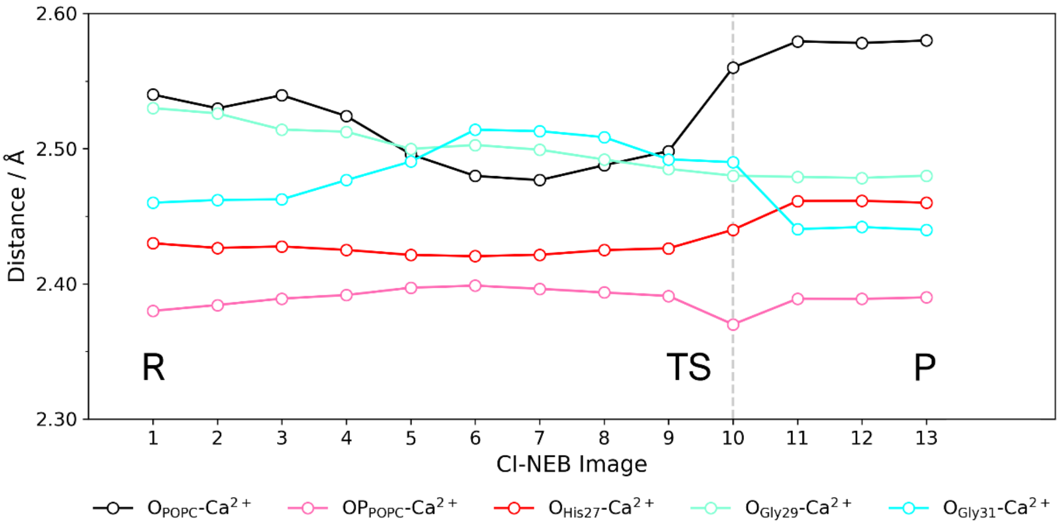

Figure S18 – Analysis of distances to the Ca^{2+} cofactor from the CI-NEB calculations of the single-water mechanism.

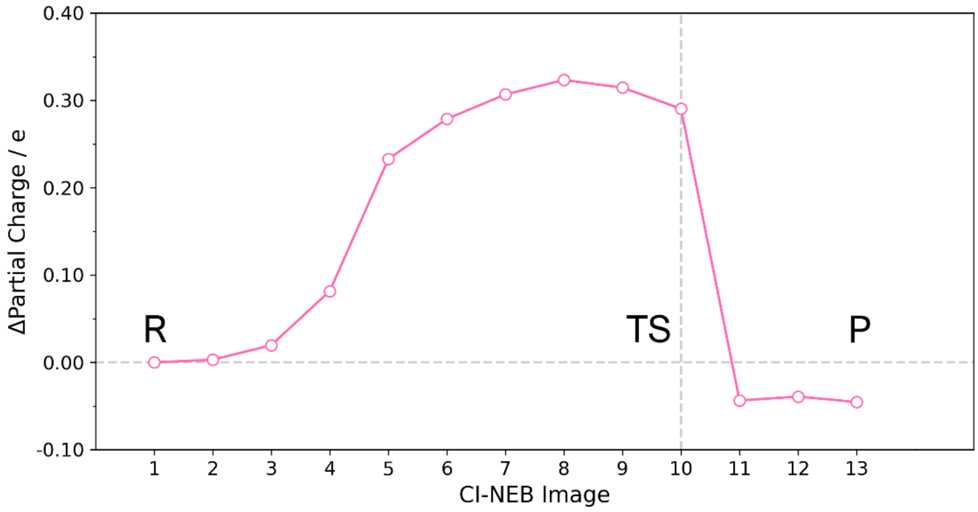

Figure S19 – ADCH charge variation of His_{47} (sidechain) from the CI-NEB calculations of the single-water mechanism.

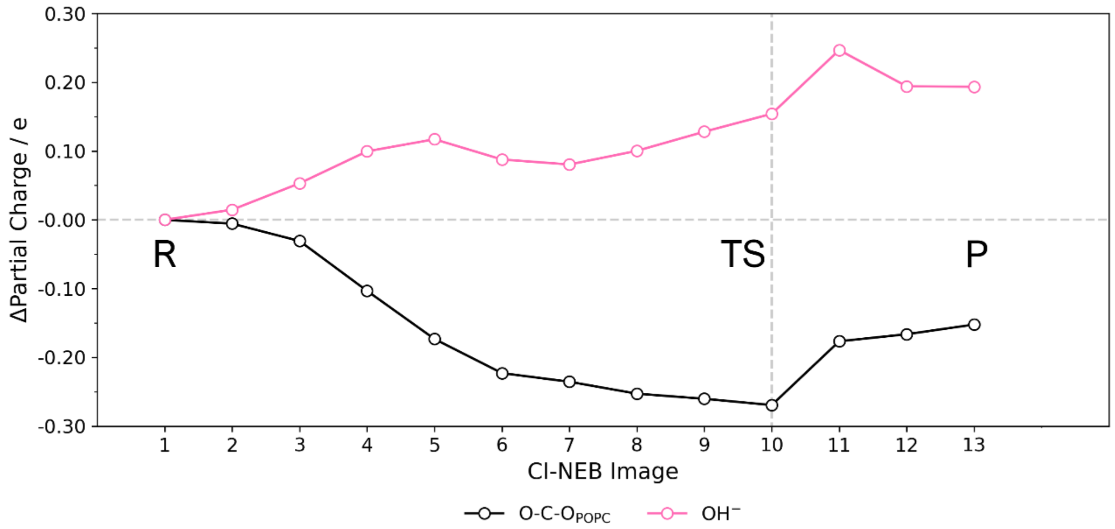

Figure S20 – ADCH charge variations of the OH^- nucleophile and the O-C-O fragment of the substrate from the CI-NEB calculations of the single-water mechanism.

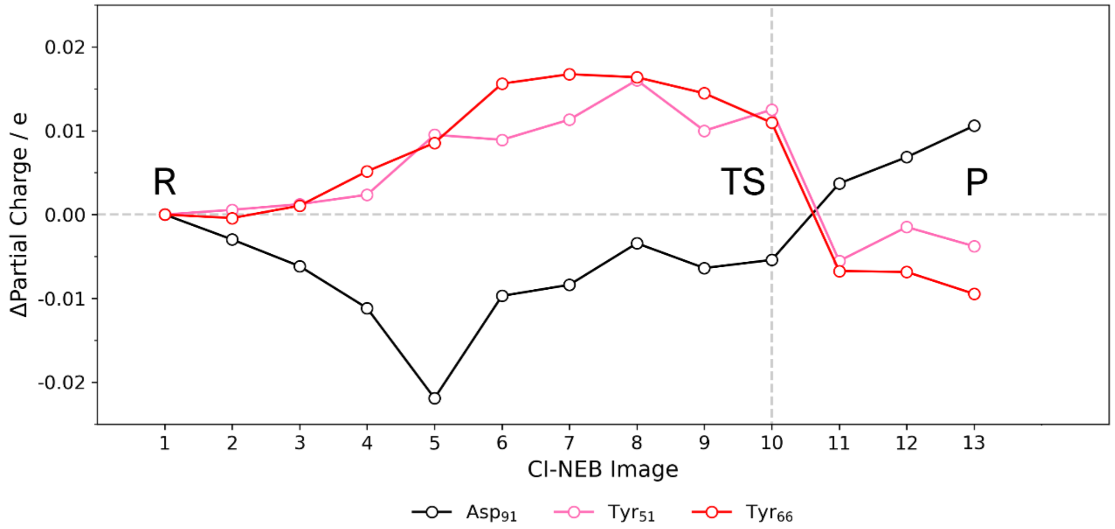

Figure S21 – ADCH charge variations of Asp_{91} (sidechain), Tyr_{51} (sidechain) and Tyr_{66} (sidechain) from the CI-NEB calculations of the single-water mechanism.

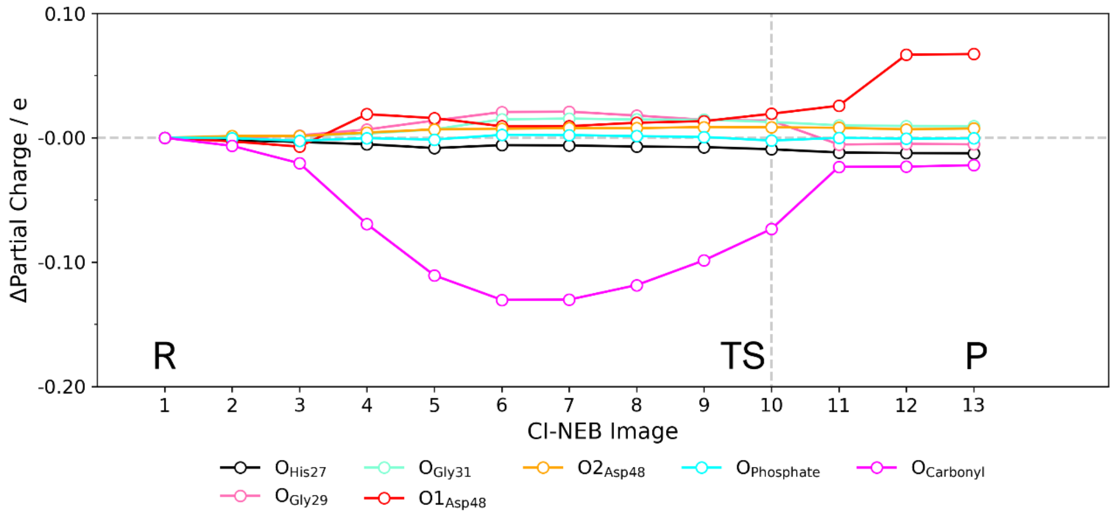

Figure S22 – ADCH charge variations of the Ca^{2+} cofactor and the oxygen atoms that coordinate it from the CI-NEB calculations of the single-water mechanism.

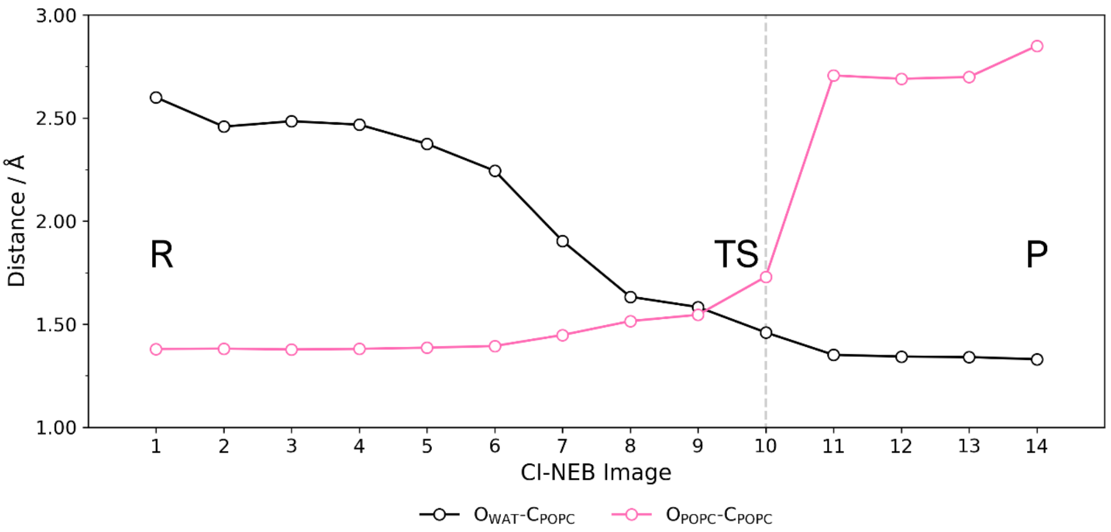

Figure S23 – Analysis of C-O bond forming (black) and C-O bond breaking (pink) distances from the CI-NEB calculations of the assisting-water mechanism.

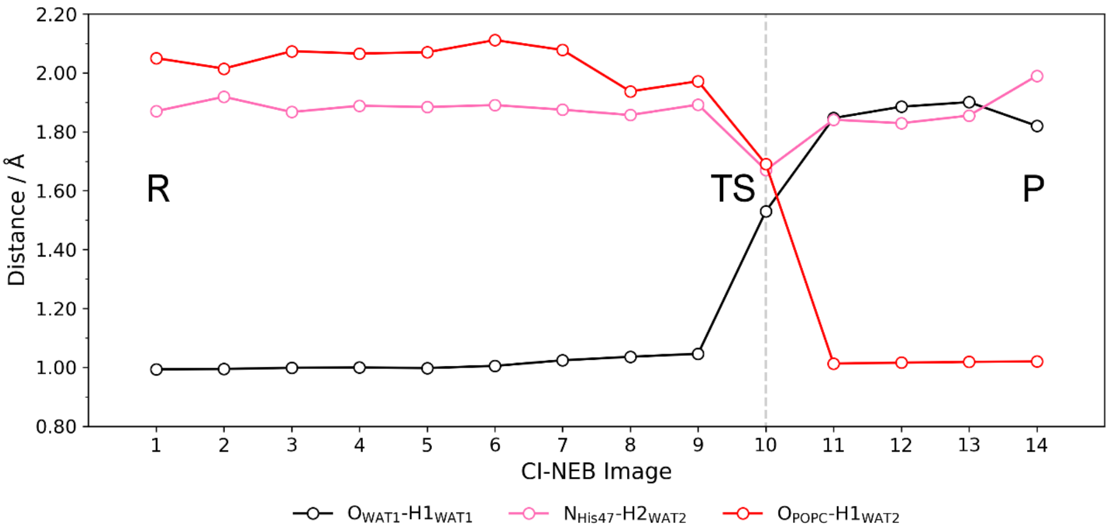

Figure S24 – Analysis of hydrogen bond forming and bond breaking distances from the CI-NEB calculations of the assisting-water mechanism.

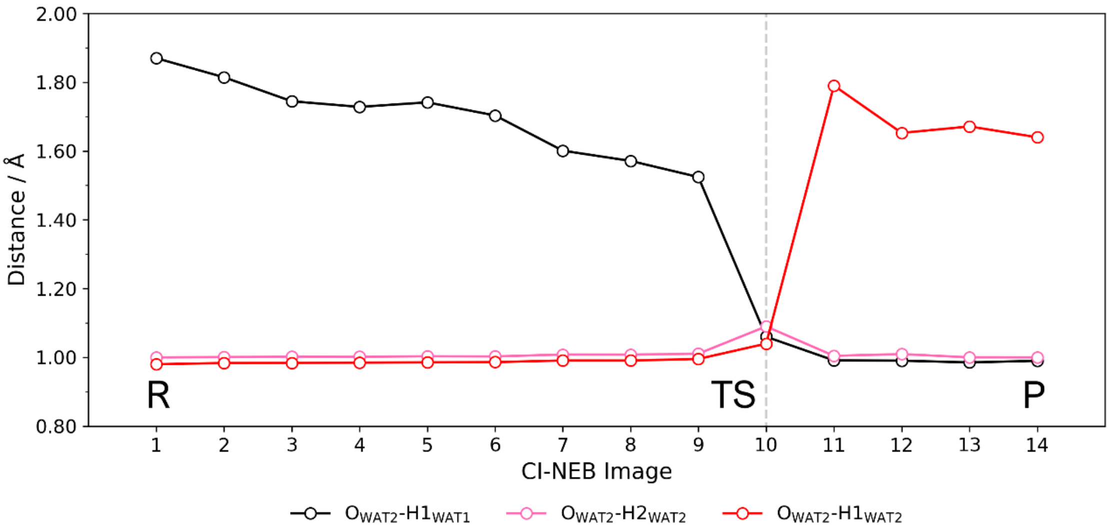

Figure S25 – Analysis of Asp_{91} hydrogen bond distances from the CI-NEB calculations of the assisting-water mechanism.

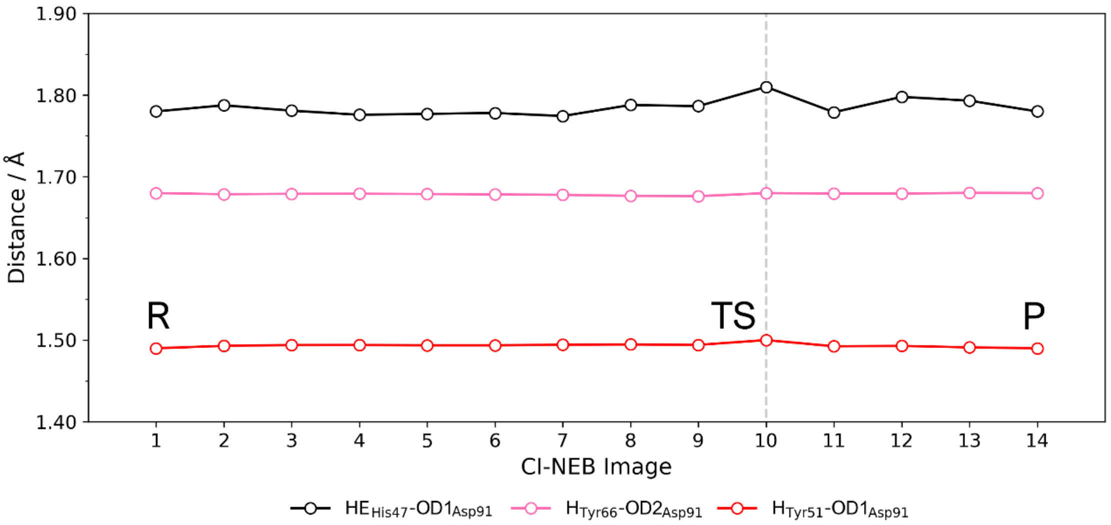

Figure S26 – Analysis of Asp_{91} hydrogen bond distances from the CI-NEB calculations of the assisting-water mechanism.

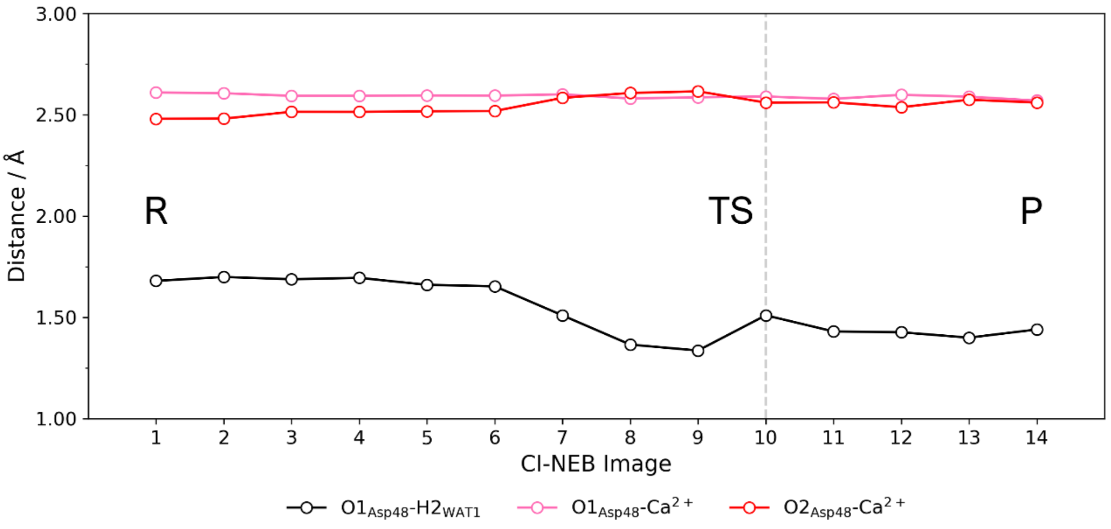

Figure S27 – Analysis of Asp_{48} distances to the Ca^{2+} cofactor and catalytic water from the CI-NEB calculations of the assisting-water mechanism.

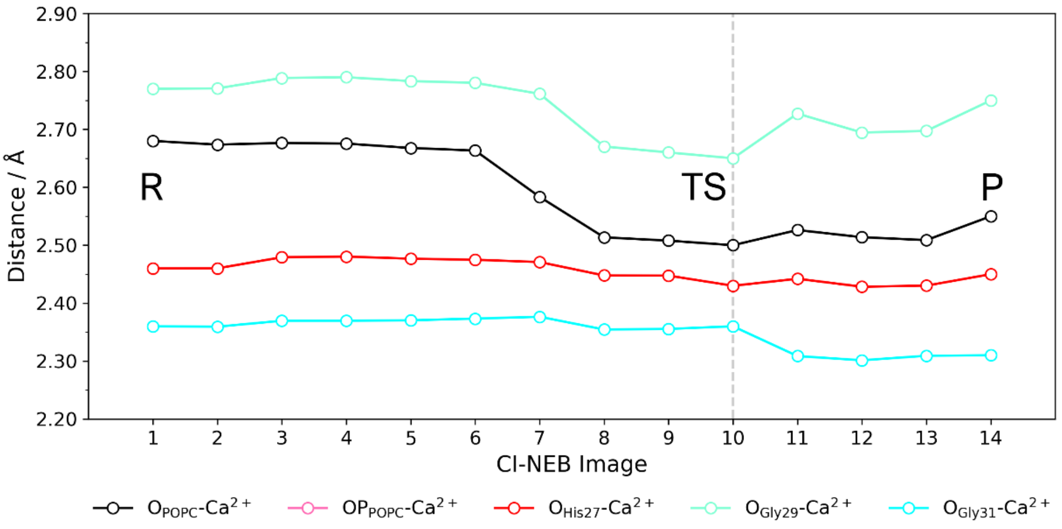

Figure S28 – Analysis of distances to the Ca^{2+} cofactor from the CI-NEB calculations of the assisting-water mechanism.

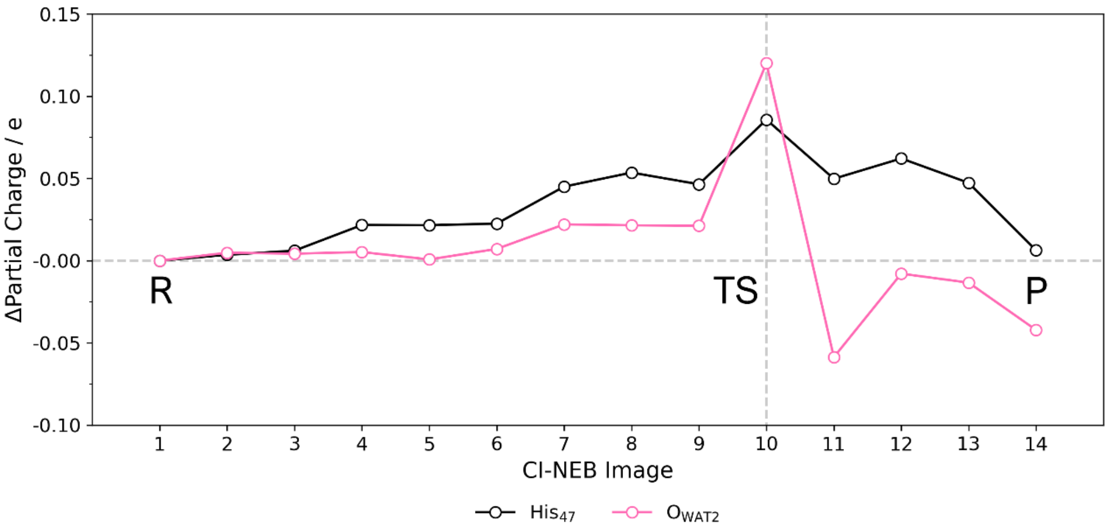

Figure S29 – ADCH charge variations of the His_{47} (sidechain) and the oxygen atom of WAT2 from the CI-NEB calculations of the assisting-water mechanism.

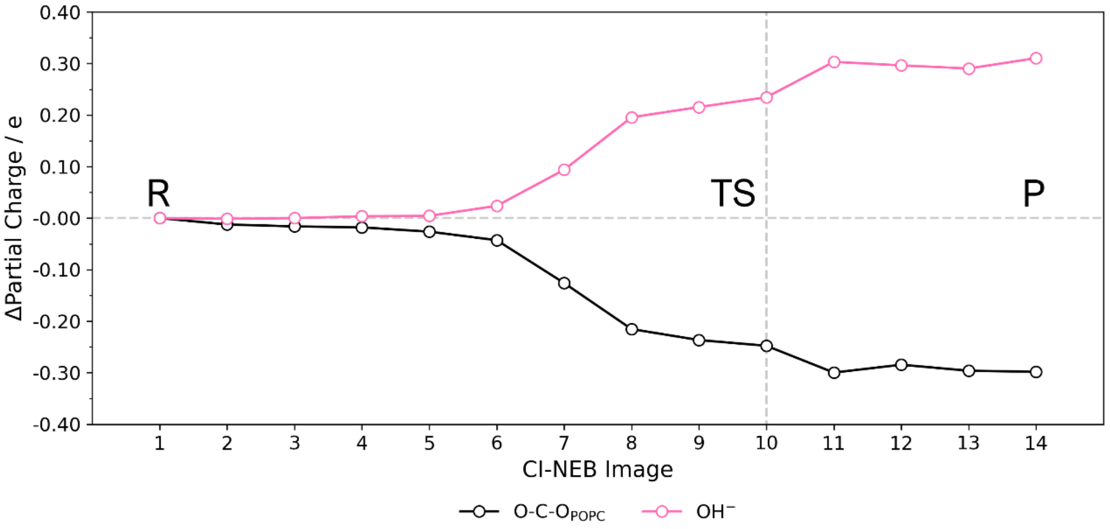

Figure S30 – ADCH charge variations of the OH^- nucleophile and the O-C-O fragment of the substrate from the CI-NEB calculations of the single-water mechanism.

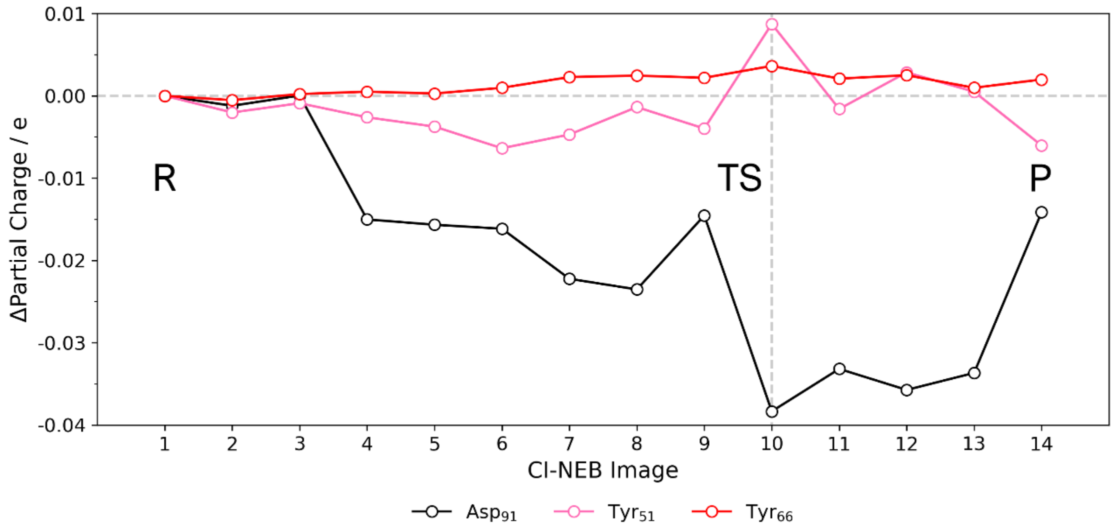

Figure S31 – ADCH charge variations of Asp_{91} (sidechain), Tyr_{51} (sidechain) and Tyr_{66} (sidechain) from the CI-NEB calculations of the assisting-water mechanism.

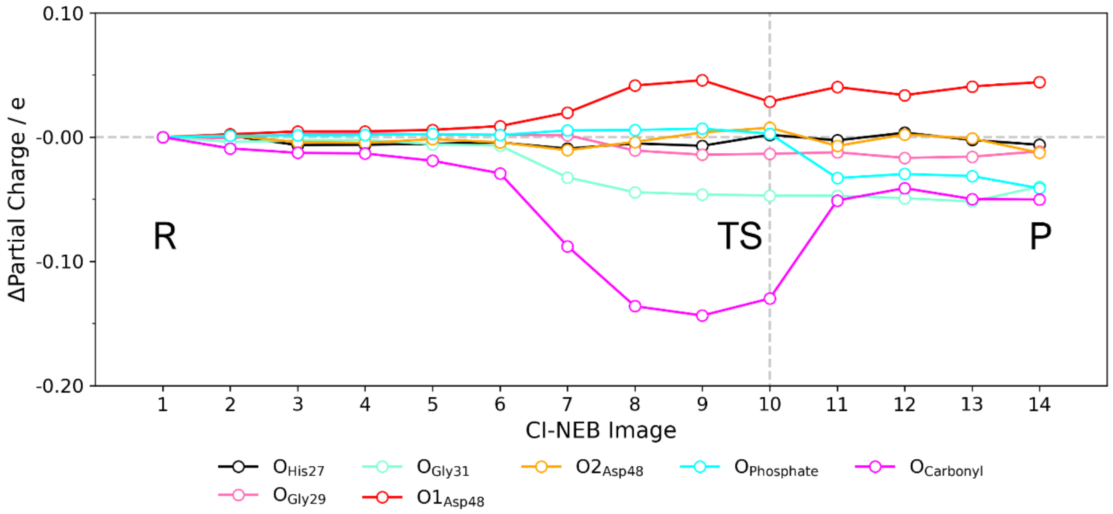

Figure S32 – ADCH charge variations of the Ca^{2+} cofactor and the oxygen atoms that coordinate it from the CI-NEB calculations of the assisting-water mechanism.

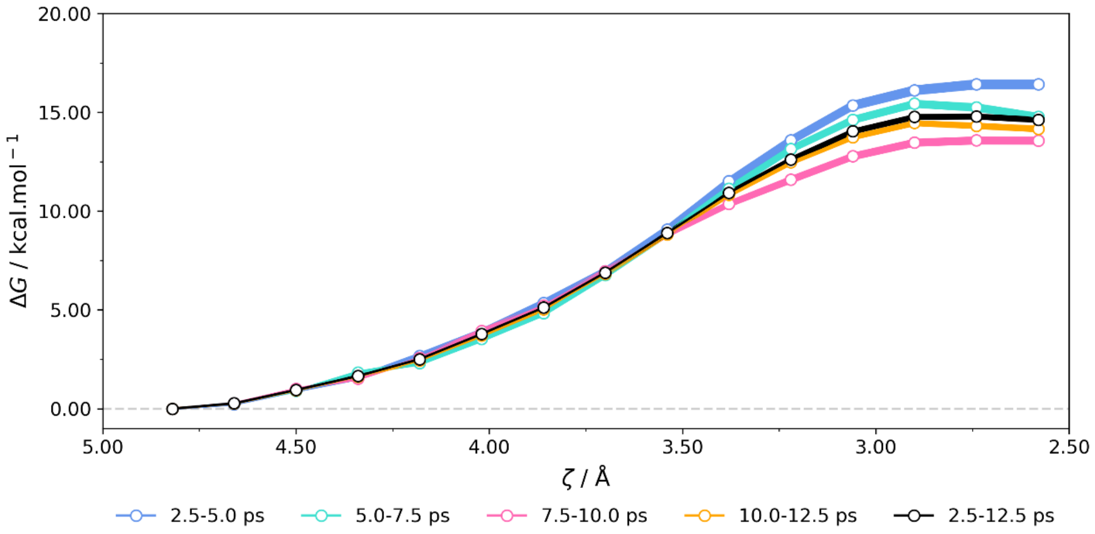

Figure S33 – Free energy curves of the CV_1 within the single-water pathway, obtained for different time blocks.

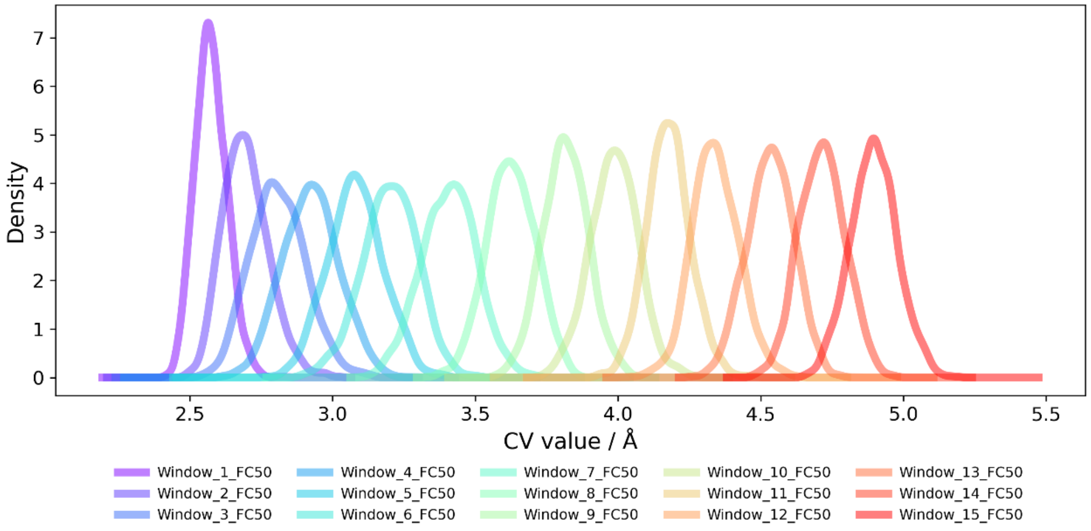

Figure S34 – Density profiles of the umbrella sampling windows, for the CV_1 within the single-water pathway.

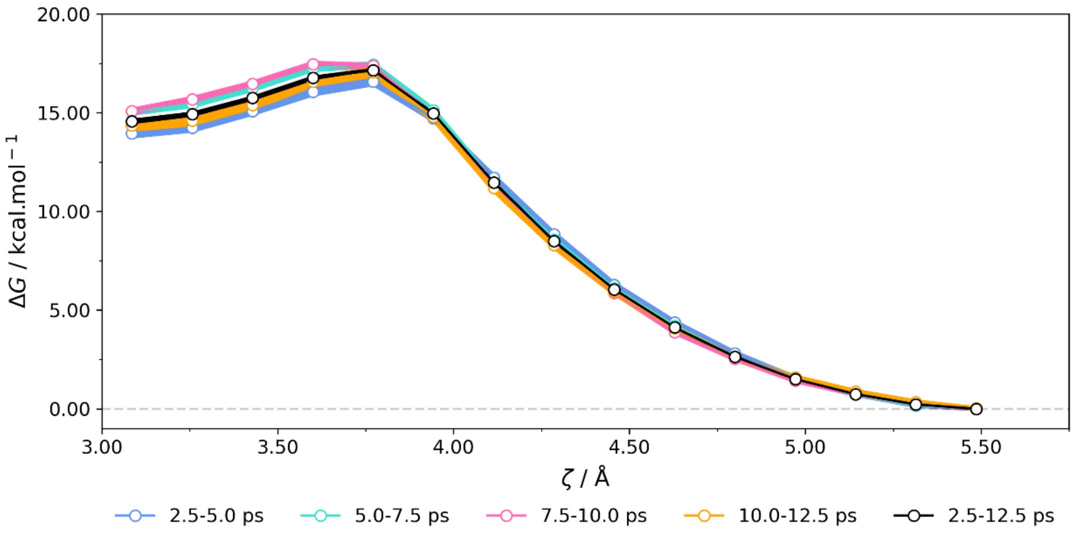

Figure S35 – Free energy curves of the CV_2 within the single-water pathway, obtained for different time blocks.

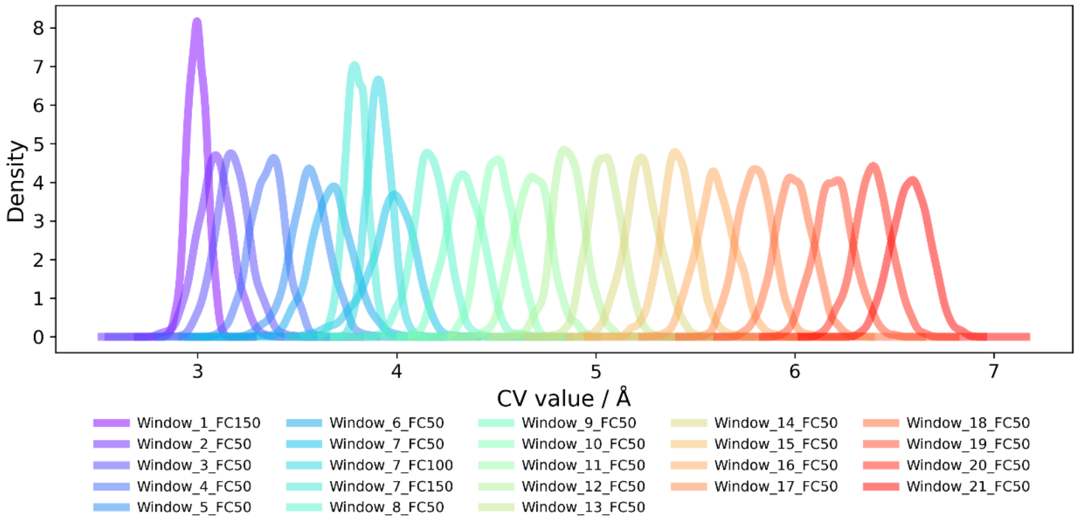

Figure S36 – Density profiles of the umbrella sampling windows, for the CV_2 within the single-water pathway.

We calculated the average 2D-RMSD of trial windows for a given reference window, considering the atoms within a given distance to the distances used in the CVs. This allowed us to evaluate if the overlapping windows belong to the same state in the free energy surface and link the free energy curves for which the reaction coordinate is regarded as a piecewise function of two different CVs.

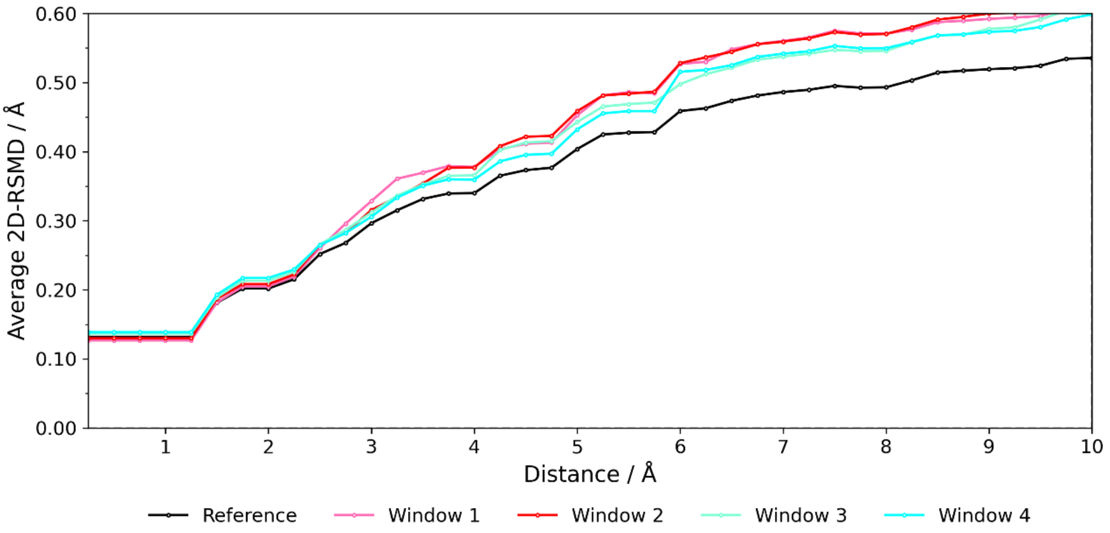

Figure S37 – Average 2D-RMSD calculated along the distance to the atoms in the CVs, using Window 14 of the first profile as a reference.

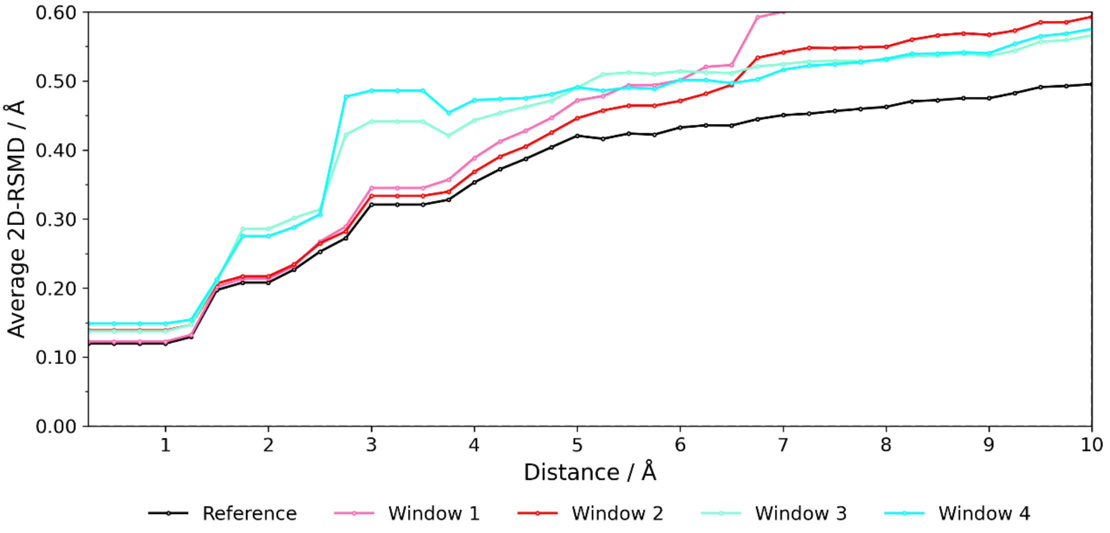

Figure S38 – Average 2D-RMSD calculated along the distance to the atoms in the CVs, using Window 2 of the second profile as a reference.

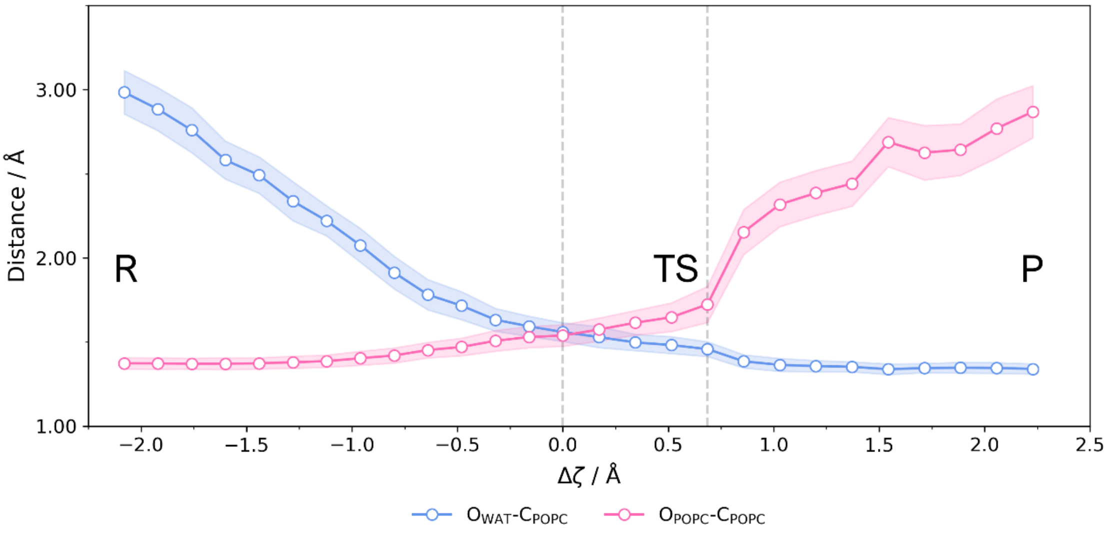

Figure S39 – Analysis of C-O bond forming (blue) and C-O bond breaking (pink) distances from the umbrella sampling calculations of the single-water mechanism. The shaded bands correspond to the standard deviation of the sampled distances.

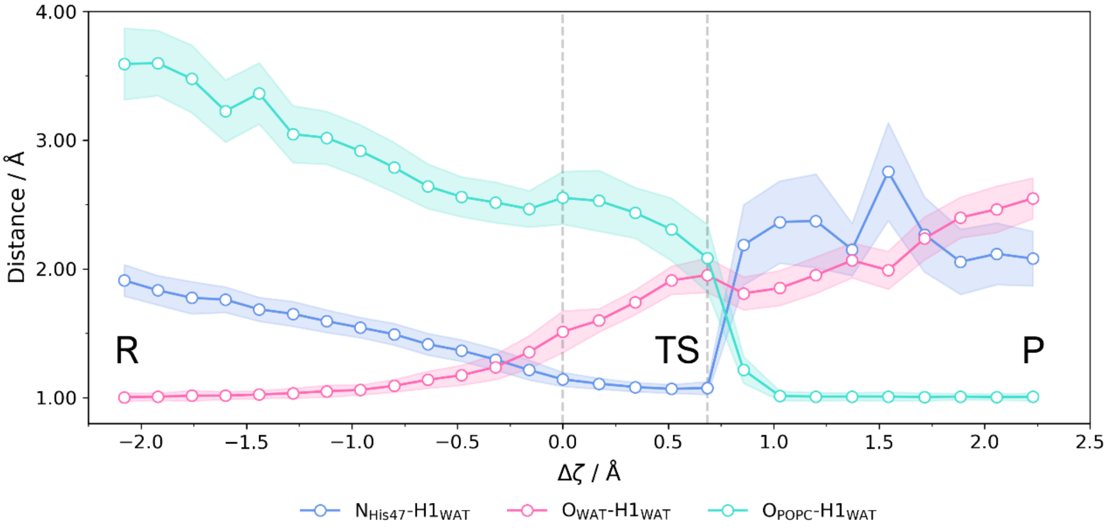

Figure S40 – Analysis of hydrogen bond forming and bond breaking distances from the umbrella sampling calculations of the single-water mechanism. The shaded bands correspond to the standard deviation of the sampled distances.

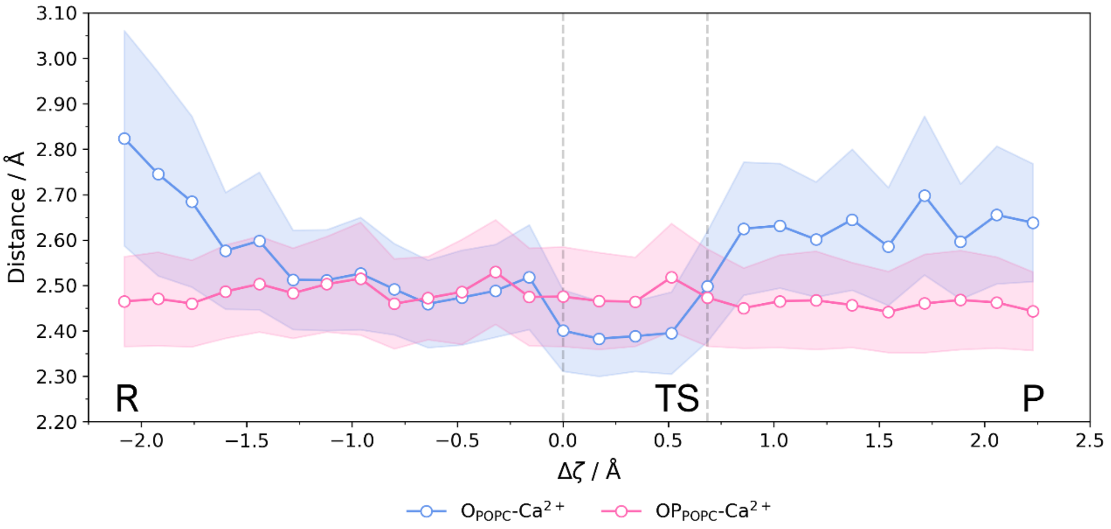

Figure S41 – Analysis of Ca^{2+} coordination distances by the oxygen atoms of the POPC substrate from the umbrella sampling calculations of the single-water mechanism. The shaded bands correspond to the standard deviation of the sampled distances.

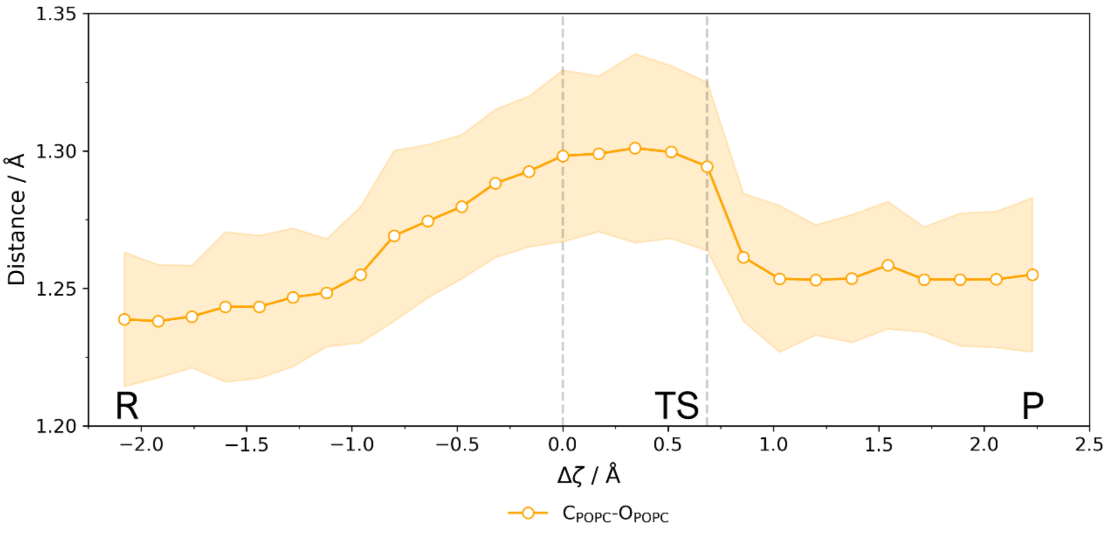

Figure S42 – Analysis of the C_{POPC}-O_{POPC} bond length from the umbrella sampling calculations of the single-water mechanism. The shaded bands correspond to the standard deviation of the sampled distances.

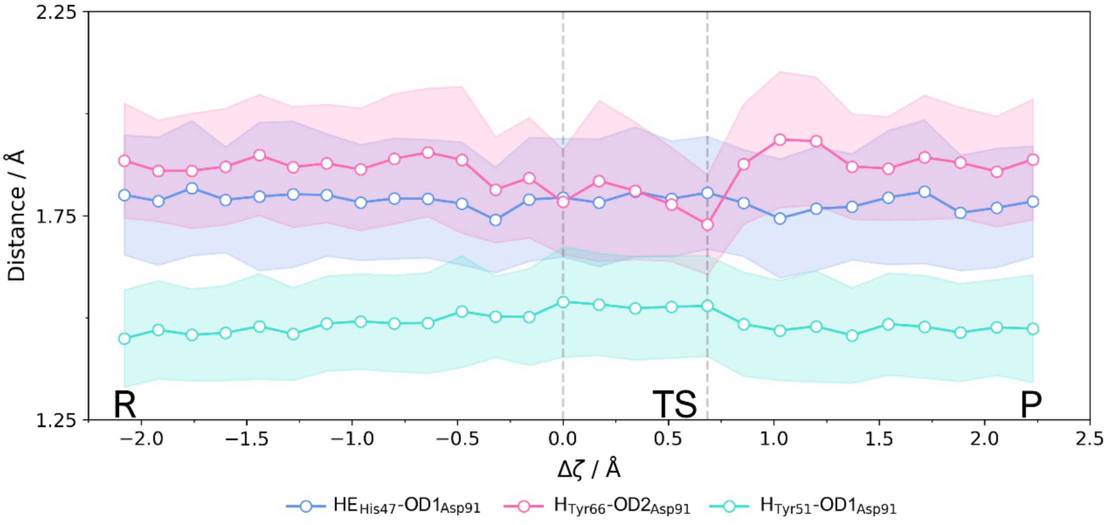

Figure S43 – Analysis of the hydrogen bond distances to Asp_{91} from the umbrella sampling calculations of the single-water mechanism. The shaded bands correspond to the standard deviation of the sampled distances.

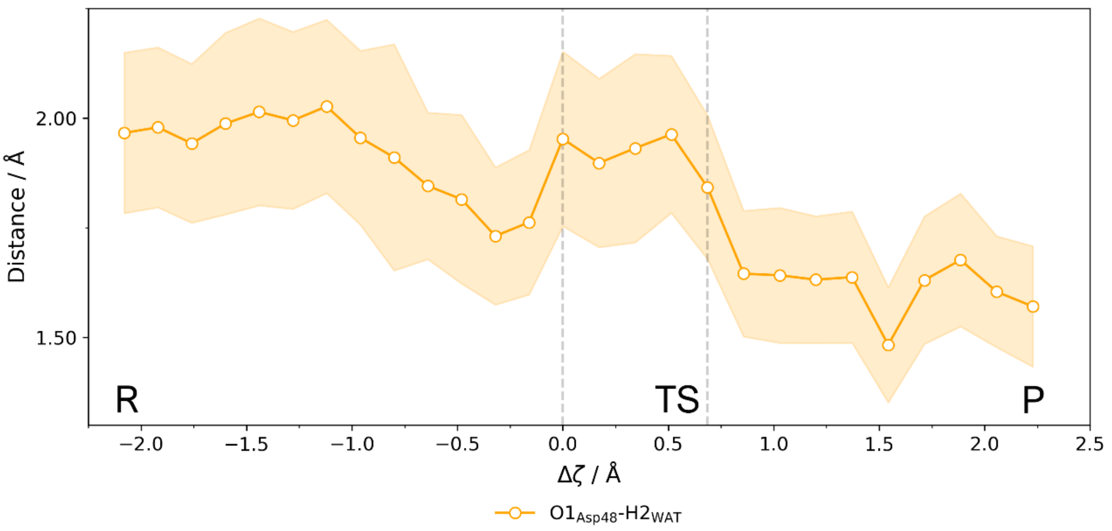

Figure S44 – Analysis of the hydrogen bond distance between the nucleophilic water and Asp_{48} from the umbrella sampling calculations of the single-water mechanism. The shaded bands correspond to the standard deviation of the sampled distances.

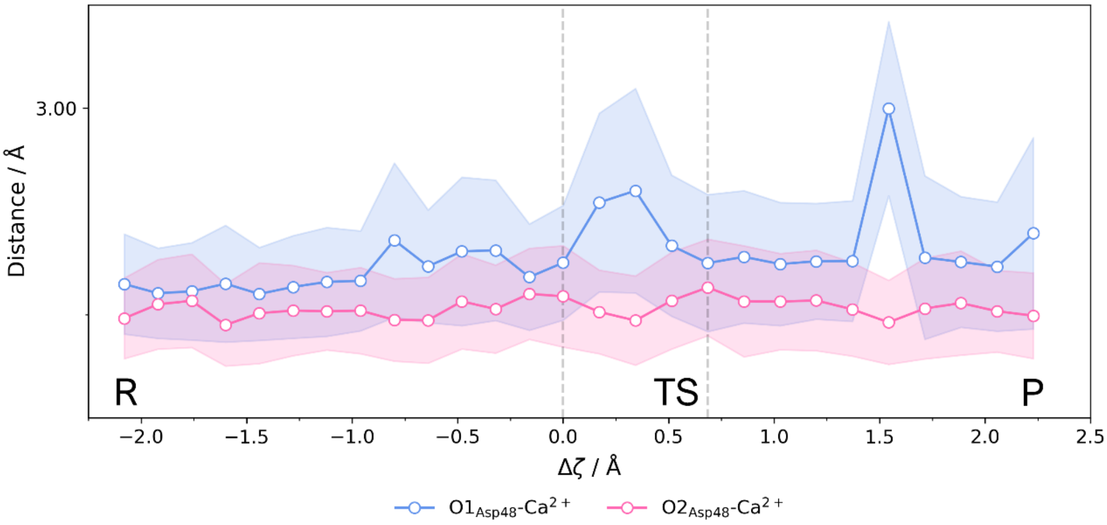

Figure S45 – Analysis of the Ca^{2+} coordination distances of Asp_{48} from the umbrella sampling calculations of the single-water mechanism. The shaded bands correspond to the standard deviation of the sampled distances.

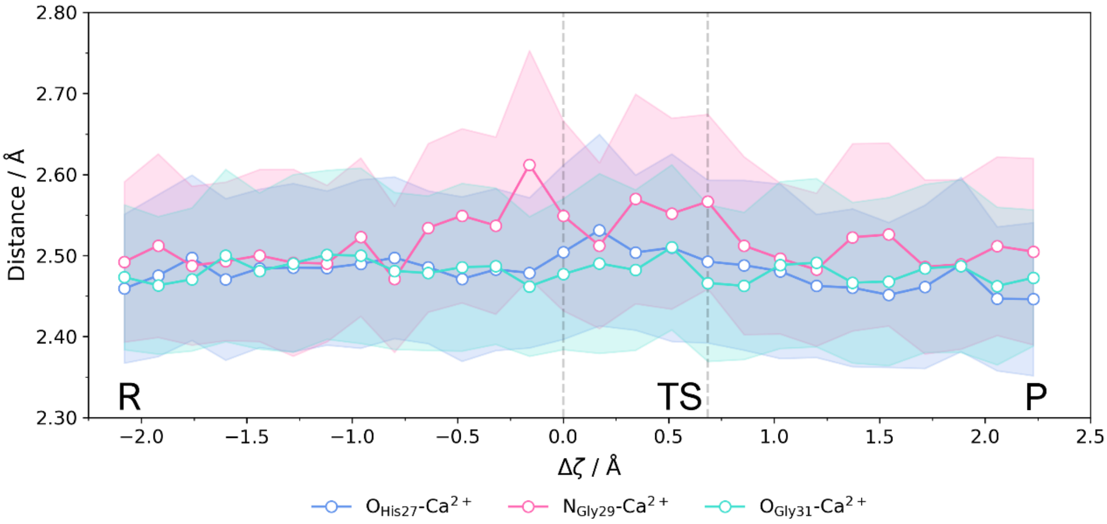

Figure S46 – Analysis of the Ca^{2+} coordination distances of the binding loop from the umbrella sampling calculations of the single-water mechanism. The shaded bands correspond to the standard deviation of the sampled distances.

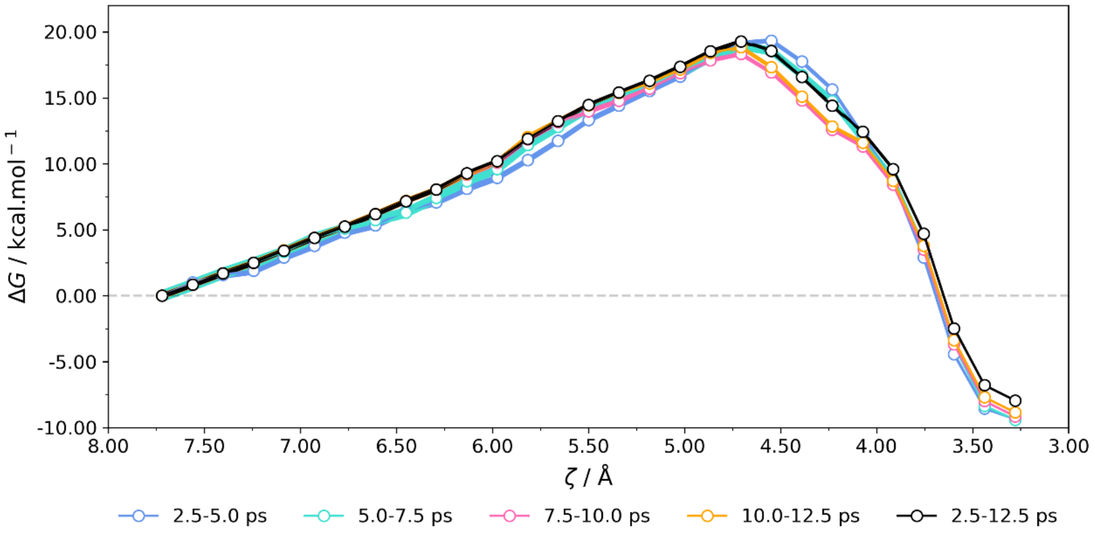

Figure S47 – Free energy curves of the assisting-water pathway, obtained for different time blocks.

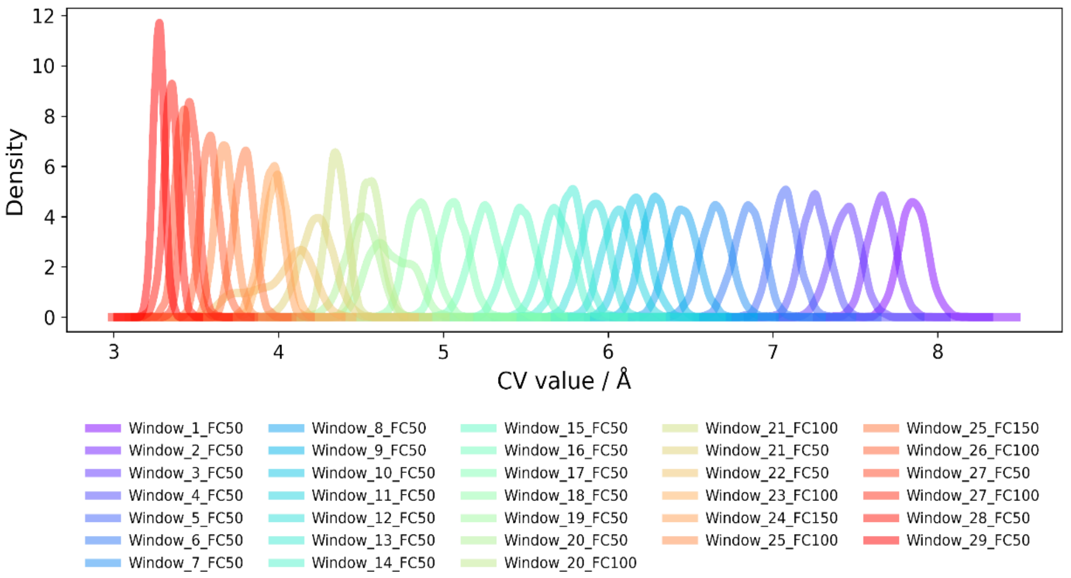

Figure S48 – Density profiles of the umbrella sampling windows for the assisting-water pathway.

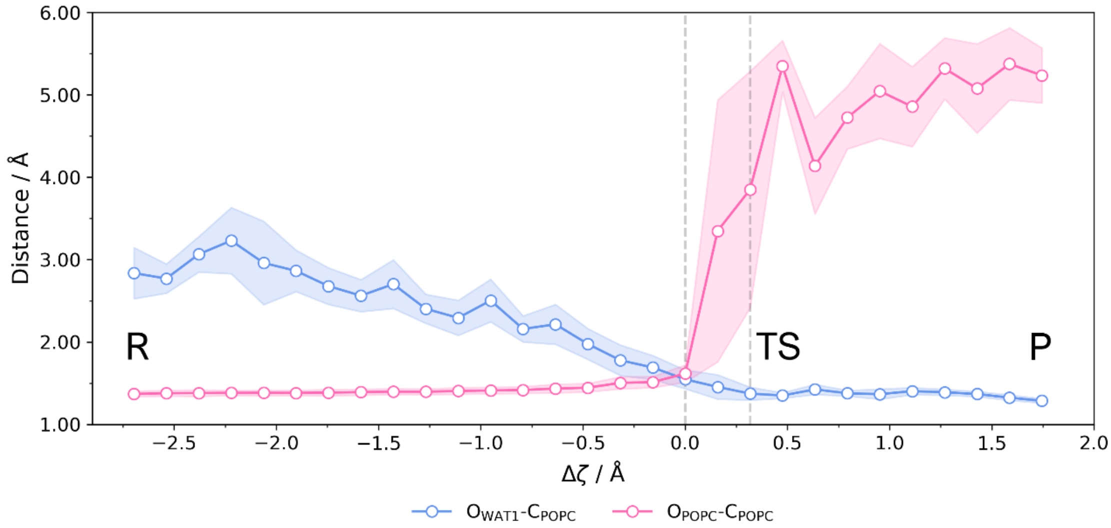

Figure S49 – Analysis of C-O bond forming (blue) and C-O bond breaking (pink) distances from the umbrella sampling calculations of the assisting-water mechanism. The shaded bands correspond to the standard deviation of the sampled distances.

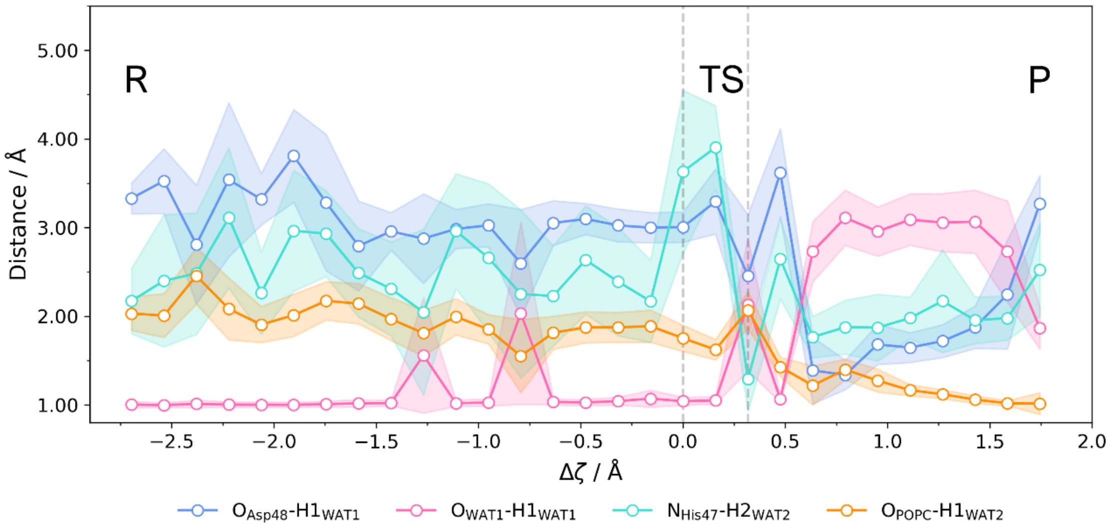

Figure S50 – Analysis of hydrogen bond forming and bond breaking distances from the umbrella sampling calculations of the assisting-water mechanism. The shaded bands correspond to the standard deviation of the sampled distances.

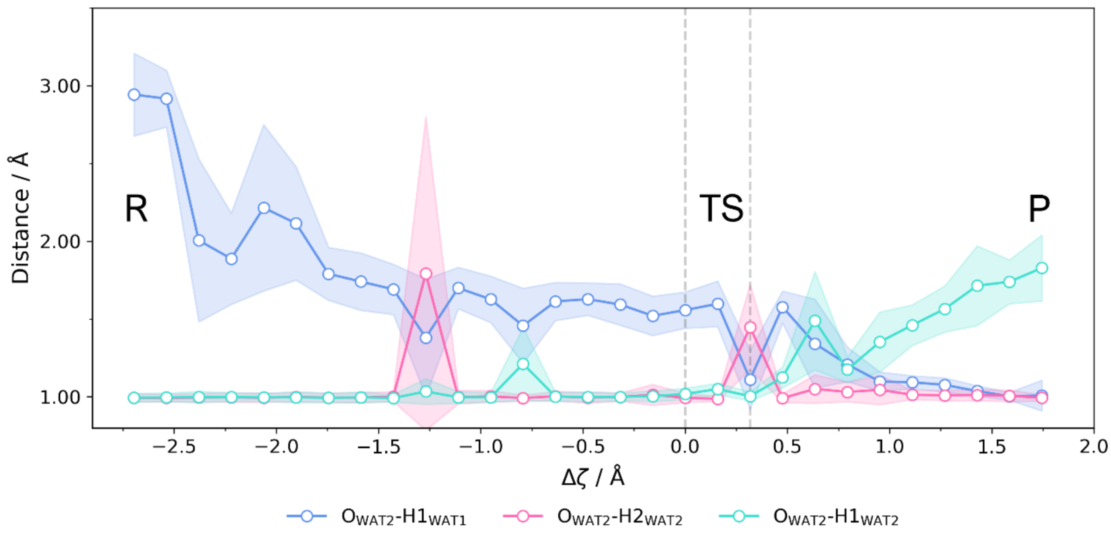

Figure S51 – Analysis of H_3O^+ distances from the umbrella sampling calculations of the assisting-water mechanism. The shaded bands correspond to the standard deviation of the sampled distances.

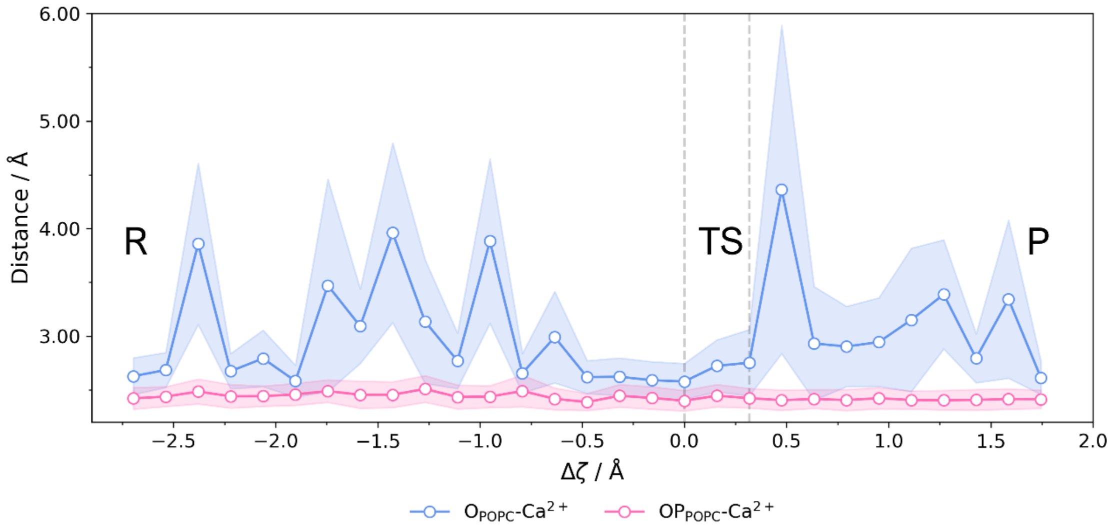

Figure S52 – Analysis of Ca^{2+} coordination distances by the oxygen atoms of the POPC substrate from the umbrella sampling calculations of the assisting-water mechanism. The shaded bands correspond to the standard deviation of the sampled distances.

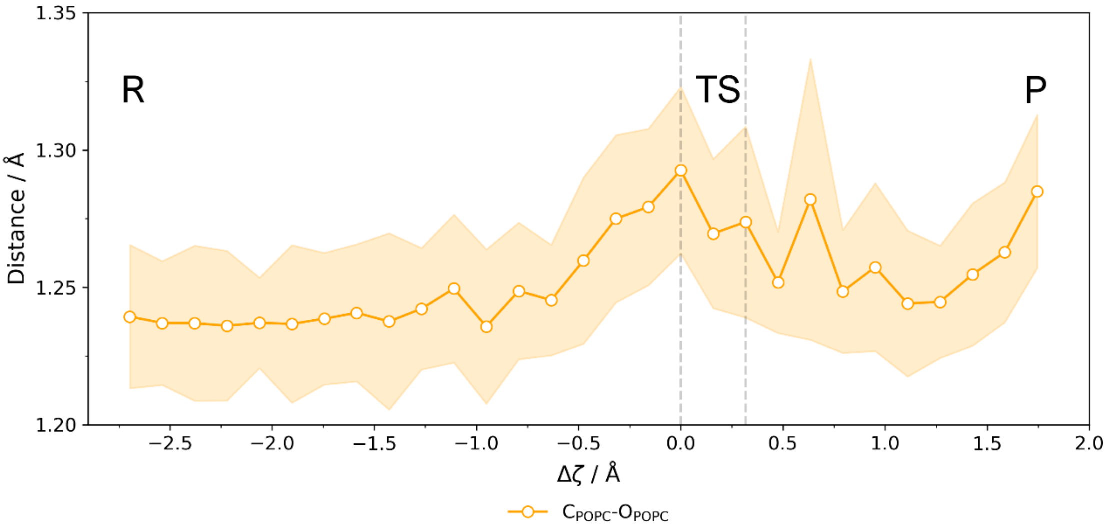

Figure S53 – Analysis of the C_{POPC}-O_{POPC} bond length from the umbrella sampling calculations of the assisting-water mechanism. The shaded bands correspond to the standard deviation of the sampled distances.

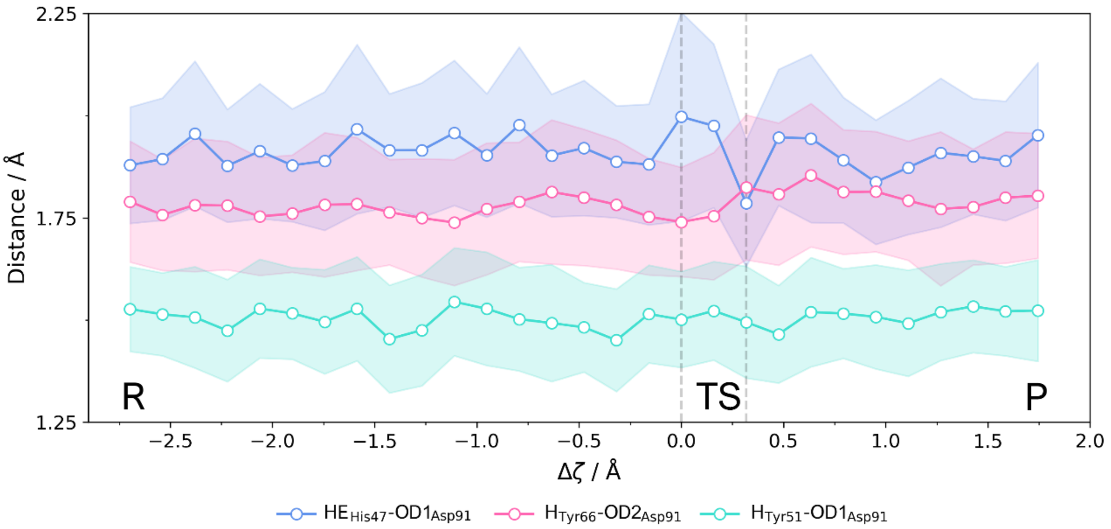

Figure S54 – Analysis of the hydrogen bond distances to Asp_{91} from the umbrella sampling calculations of the assisting-water mechanism. The shaded bands correspond to the standard deviation of the sampled distances.

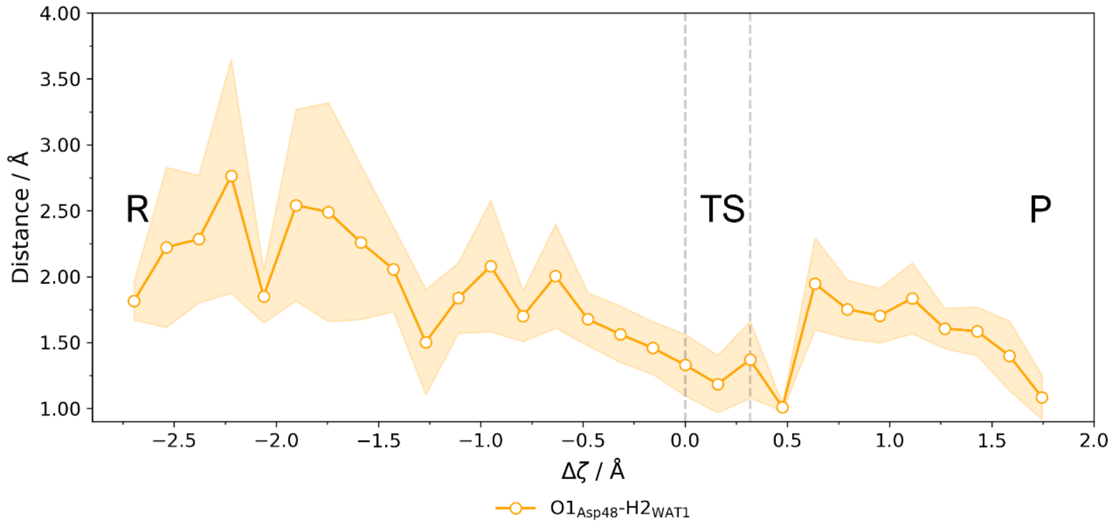

Figure S55 – Analysis of the hydrogen bond distance between the nucleophilic water and Asp_{48} from the umbrella sampling calculations of the assisting-water mechanism. The shaded bands correspond to the standard deviation of the sampled distances.

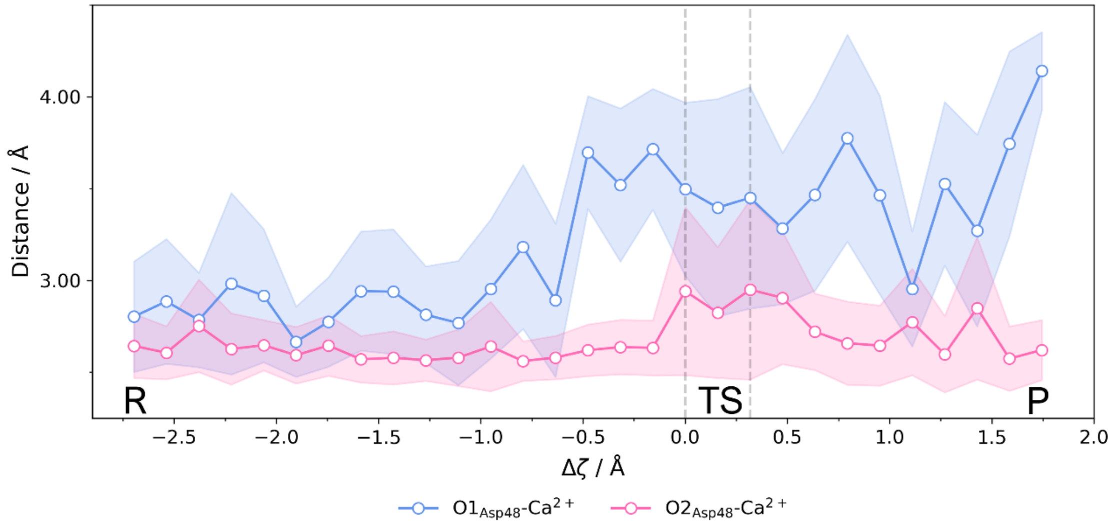

Figure S56 – Analysis of the Ca^{2+} coordination distances of Asp_{48} from the umbrella sampling calculations of the assisting-water mechanism. The shaded bands correspond to the standard deviation of the sampled distances.

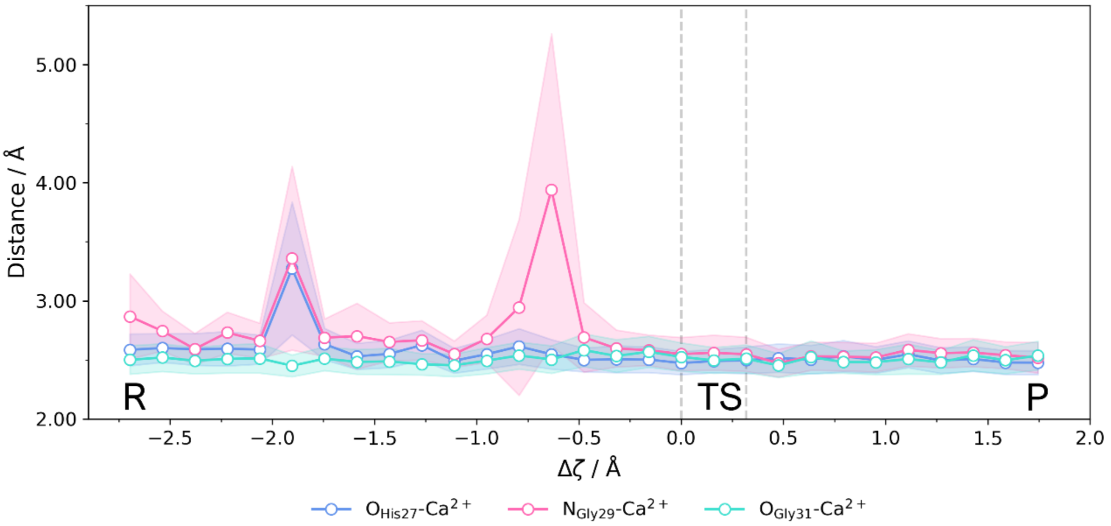

Figure S57 – Analysis of the Ca^{2+} coordination distances of the binding loop from the umbrella sampling calculations of the assisting-water mechanism. The shaded bands correspond to the standard deviation of the sampled distances.

Table S1 – Per residue energy contributions (residues 1-65) to the activation energy for both reaction pathways.

Number	Residue	Single-Water		Assisting-Water		Number	Residue	Single-Water		Assisting-Water	
**1**	**ASN**	0.00	1.10	-2.26	-0.59	**35**	**SER**	1.95	1.51	1.34	1.02
**2**	**LEU**	-2.04	1.91	-1.43	1.38	**36**	**PRO**	2.17	1.77	1.31	1.24
**3**	**VAL**	-1.17	1.63	-1.62	1.14	**37**	**LYS**	2.26	2.42	1.85	1.80
**4**	**ASN**	-1.17	2.46	-2.24	0.69	**38**	**ASP**	2.36	1.41	1.54	0.73
**5**	**PHE**	-2.07	2.37	-1.65	1.85	**39**	**ALA**	2.13	1.72	1.09	1.14
**6**	**HIE**	-1.65	1.61	-0.78	1.14	**40**	**THR**	1.91	1.83	1.32	1.12
**7**	**ARG**	-0.30	1.81	-1.32	1.30	**41**	**ASP**	1.85	0.66	1.60	-0.75
**8**	**MET**	-1.38	2.42	-1.74	1.03	**42**	**ARG**	2.30	1.86	0.86	1.35
**9**	**ILE**	-1.83	1.79	-0.52	1.21	**43**	**CYX**	2.08	3.12	0.49	1.67
**10**	**LYS**	-0.24	2.55	-0.52	1.40	**44**	**CYX**	1.27	2.13	1.16	2.04
**11**	**LEU**	-0.12	1.67	-1.06	1.10	**45**	**VAL**	2.21	2.38	0.61	1.81
**12**	**THR**	-1.08	1.67	-0.65	1.12	**-**	**-**	-	-	-	-
**13**	**THR**	-0.73	1.53	0.13	1.01	**50**	**CYX**	-0.12	1.05	-0.96	0.38
**14**	**GLY**	0.33	1.66	-0.05	1.13	**-**	**-**	-	-	-	-
**15**	**LYS**	0.42	2.10	0.54	1.55	**52**	**LYS**	-2.13	1.42	-0.09	0.33
**16**	**GLU**	0.96	1.30	0.30	0.77	**53**	**ARG**	-0.66	0.15	-0.47	-0.25
**17**	**ALA**	0.60	1.44	0.68	1.03	**54**	**LEU**	-1.52	1.53	-1.25	0.86
**18**	**ALA**	0.74	1.38	1.19	0.99	**55**	**GLU**	-2.23	1.67	-0.47	2.14
**19**	**LEU**	1.16	1.62	1.51	1.02	**56**	**LYS**	-1.40	1.43	-0.35	0.47
**20**	**SER**	1.65	1.84	1.42	1.12	**57**	**ARG**	-1.24	0.97	-0.99	-0.04
**21**	**TYR**	1.72	1.76	1.34	0.62	**58**	**GLY**	-2.12	1.67	-0.99	1.14
**22**	**GLY**	1.51	1.20	2.08	0.94	**59**	**CYX**	-2.13	1.61	-1.43	1.20
**23**	**PHE**	1.99	1.56	2.24	1.09	**60**	**GLY**	-2.43	1.82	-1.71	1.27
**24**	**TYR**	2.15	1.75	2.06	1.24	**61**	**THR**	-2.48	1.56	-1.83	0.68
**25**	**GLY**	2.48	1.26	2.22	1.04	**62**	**LYS**	-2.26	4.21	-1.23	1.26
**26**	**CYX**	2.64	1.88	1.98	1.17	**63**	**PHE**	-1.50	1.69	-1.77	1.13
**-**	**-**	-	-	-	-	**64**	**LEU**	-1.55	1.70	-2.14	1.22
**33**	**ARG**	1.51	2.61	1.51	2.32	**65**	**SER**	-2.68	1.71	-2.37	1.17
**34**	**GLY**	1.84	1.82	1.34	1.14	**-**	**-**	-	-	-	-

Table S2 – Per residue energy contributions (residues 67-124) to the activation energy for both reaction pathways.

Number	Residue	Single-Water		Assisting-Water		Number	Residue	Single-Water		Assisting-Water	
**67**	**LYS**	-2.82	1.38	-2.48	0.63	**96**	**THR**	-0.92	1.58	-0.84	1.02
**68**	**PHE**	-2.78	1.64	-2.28	1.16	**97**	**CYX**	-0.81	1.50	-0.02	0.90
**69**	**SER**	-2.42	1.75	-2.19	1.22	**98**	**PHE**	0.32	1.55	0.37	1.14
**70**	**ASN**	-2.28	1.75	-1.67	1.18	**99**	**ALA**	0.67	1.57	0.00	1.08
**71**	**SER**	-1.80	1.74	-1.57	1.21	**100**	**ARG**	0.08	0.70	0.01	0.43
**72**	**GLY**	-1.93	1.65	-1.19	1.18	**101**	**ASN**	0.43	1.72	0.78	1.11
**73**	**SER**	-1.53	1.68	-1.05	1.21	**102**	**LYS**	1.11	1.79	0.64	1.10
**74**	**ARG**	-1.18	0.37	-1.53	0.71	**103**	**THR**	0.88	1.65	1.00	1.12
**75**	**ILE**	-1.26	1.71	-1.74	1.15	**104**	**THR**	1.21	1.68	1.21	1.11
**76**	**THR**	-1.91	1.68	-2.17	1.14	**105**	**TYR**	1.58	1.81	1.63	1.28
**77**	**CYX**	-2.32	1.75	-2.27	1.14	**106**	**ASN**	1.82	1.61	1.89	1.04
**78**	**ALA**	-2.68	1.74	-2.38	1.22	**107**	**LYS**	2.18	1.98	1.98	1.51
**79**	**LYS**	-2.84	1.10	-2.31	0.52	**108**	**LYS**	2.13	2.01	2.16	1.64
**80**	**GLN**	-2.88	1.61	-2.34	1.11	**109**	**TYR**	2.53	1.58	2.09	1.03
**81**	**ASP**	-2.97	2.11	-2.13	1.97	**110**	**GLN**	2.53	1.71	2.13	1.07
**82**	**SER**	-2.94	1.74	-1.89	1.25	**111**	**TYR**	2.28	1.64	2.29	1.16
**83**	**CYX**	-2.85	1.74	-1.88	1.25	**112**	**TYR**	2.37	1.53	2.28	0.98
**84**	**ARG**	-2.79	1.39	-2.29	0.55	**113**	**SER**	2.64	1.72	2.31	1.23
**85**	**SER**	-2.93	1.84	-2.16	1.23	**114**	**ASN**	2.52	1.82	2.03	1.05
**86**	**GLN**	-2.92	1.93	-1.80	1.42	**115**	**LYS**	2.29	2.73	2.14	2.03
**87**	**LEU**	-2.71	1.38	-2.29	1.27	**116**	**HID**	2.29	2.03	2.19	1.28
**88**	**CYX**	-2.96	1.90	-2.33	1.42	**117**	**CYX**	2.50	1.71	1.95	1.16
**89**	**GLU**	-2.85	3.73	-1.93	2.39	**118**	**ARG**	2.30	2.23	1.81	1.65
**90**	**CYX**	-2.54	2.29	-1.81	1.16	**119**	**GLY**	2.18	1.65	1.33	1.04
**-**	**-**	-	-	-	-	**120**	**SER**	2.02	1.73	1.21	1.18
**92**	**LYS**	-2.71	-0.39	-1.87	-0.20	**121**	**THR**	1.87	1.70	1.18	1.25
**93**	**ALA**	-2.05	1.43	-1.33	0.87	**122**	**PRO**	1.64	1.63	0.92	1.14
**94**	**ALA**	-1.44	1.51	-0.82	0.70	**123**	**ARG**	1.66	1.64	0.85	1.42
**95**	**ALA**	-0.85	1.66	-0.85	1.06	**124**	**CYX**	1.05	2.72	0.29	1.67
